# Neuronal uptake and propagation of a rare phosphorylated high-molecular-weight tau derived from Alzheimer’s disease brain

**DOI:** 10.1038/ncomms9490

**Published:** 2015-10-13

**Authors:** Shuko Takeda, Susanne Wegmann, Hansang Cho, Sarah L. DeVos, Caitlin Commins, Allyson D. Roe, Samantha B. Nicholls, George A. Carlson, Rose Pitstick, Chloe K. Nobuhara, Isabel Costantino, Matthew P. Frosch, Daniel J. Müller, Daniel Irimia, Bradley T. Hyman

**Affiliations:** 1 Department of Neurology, Alzheimer’s Disease Research Laboratory, MassGeneral Institute for Neurodegenerative Disease, Massachusetts General Hospital, Harvard Medical School, Charlestown, Massachusetts 02129, USA; 2 BioMEMS Resource Center, Massachusetts General Hospital, Harvard Medical School, Charlestown, Massachusetts 02129, USA; 3 Department of Mechanical Engineering and Engineering Science, University of North Carolina at Charlotte, Charlotte, North Carolina 28223, USA; 4 McLaughlin Research Institute, Great Falls, Montana 59405, USA; 5 Department of Biosystems Science and Engineering, Eidgenössische Technische Hochschule Zürich, 4058 Basel, Switzerland

## Abstract

Tau pathology is known to spread in a hierarchical pattern in Alzheimer’s disease (AD) brain during disease progression, likely by trans-synaptic tau transfer between neurons. However, the tau species involved in inter-neuron propagation remains unclear. To identify tau species responsible for propagation, we examined uptake and propagation properties of different tau species derived from postmortem cortical extracts and brain interstitial fluid of tau-transgenic mice, as well as human AD cortices. Here we show that PBS-soluble phosphorylated high-molecular-weight (HMW) tau, though very low in abundance, is taken up, axonally transported, and passed on to synaptically connected neurons. Our findings suggest that a rare species of soluble phosphorylated HMW tau is the endogenous form of tau involved in propagation and could be a target for therapeutic intervention and biomarker development.

## Abstract (web summary)

In Alzheimer's disease, tau spreads throughout the brain, however the nature of the tau species propagating from one neuron to another is not known. Here, Takeda *et al*. identify a rare, high-molecular-weight tau as the primary species taken up and transferred between synaptically connected neurons.

## Graphical abstract

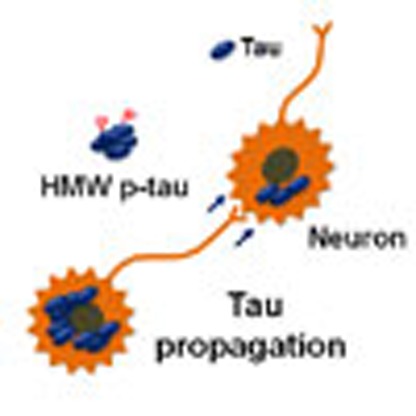

Accumulation and aggregation of microtubule-associated protein tau^1^, as intracellular inclusions known as neurofibrillary tangles (NFTs), is a pathological hallmark of neurodegenerative diseases including Alzheimer’s disease (AD)^2^,^3^. Cognitive deficits in AD are most closely linked with progression of NFTs in a hierarchical pattern, starting in the entorhinal cortex (EC) and marching throughout the brain during disease progression^4^,^5^. Although the precise mechanisms for this characteristic tau pathology spread remain unknown, accumulating evidence suggests a trans-synaptic transfer of tau proteins between neurons^6^,^7^,^8^. By developing the rTgTauEC mouse model of early AD that overexpresses human mutant P301L tau selectively in the EC, we and other groups have demonstrated that aggregated tau accumulates in synaptically connected downstream areas such as dentate gyrus, suggesting that NFT propagation occurs by cross-synaptic spread of pathologically misfolded tau proteins^9^,^10^,^11^,^12^. Other studies demonstrated that pathological forms of tau replicate conformation and spread among cells, thus suggesting that prion-like mechanisms underlie the stereotyped propagation of tau^13^,^14^,^15^,^16^. It has been shown that tau can be secreted from intact neurons into the extracellular space in an activity-dependent manner^17^,^18^, supporting the idea that extracellular misfolded tau that is taken up by neurons may provide a platform for tau pathology spreading. Better understanding of the molecular basis of tau propagation is key to preventing progression from early mild memory impairment to full cognitive deterioration and dementia.

Recent studies showed that cellular tau uptake and trans-cellular propagation occur in various systems *in vitro* and *in vivo*; however, whether the tau species involved in neuron-to-neuron transfer is fibrillar or not, and what its specific properties are, remains uncertain. In this study, to identify the specific tau species responsible for propagation, we compared the uptake and propagation properties of different tau species derived from brain extracts of tau-transgenic mouse lines rTg4510 (expressing aggregating P301L tau (0N4R))^19^ and rTg21221 (expressing non-aggregating wild-type (WT) human tau (0N4R))^20^, human sporadic AD brain extracts, and recombinant WT full-length human tau (2N4R, 441 amino acid (aa)). We isolated the propagating tau species via differential centrifugation and size-exclusion chromatography (SEC), characterized it biochemically, and then studied its neuronal uptake in mouse primary cortical neurons and *in vivo*. For all different sources of tau, efficient uptake was only observed for high-molecular-weight (HMW) tau species.

We then examined the transfer of tau between neurons using a newly developed microfluidic neuron culture platform, which comprises three distinct chambers that are connected through arrays of thin channels such that the axon growth and formation of synaptic connections are precisely controlled between neurons in different chambers. Furthermore, a unique large-pore (1,000 kDa cutoff) probe *in vivo* microdialysis^21^,^22^ allowed us to investigate the presence of HMW tau species in brain interstitial fluid (ISF) of awake, freely moving mice. Our findings suggest that PBS-soluble phosphorylated HMW tau species, present in the brain extracellular space, are involved in neuronal uptake and propagation.

## Results

### Identification of tau species taken up by neurons

**Figure 1:**
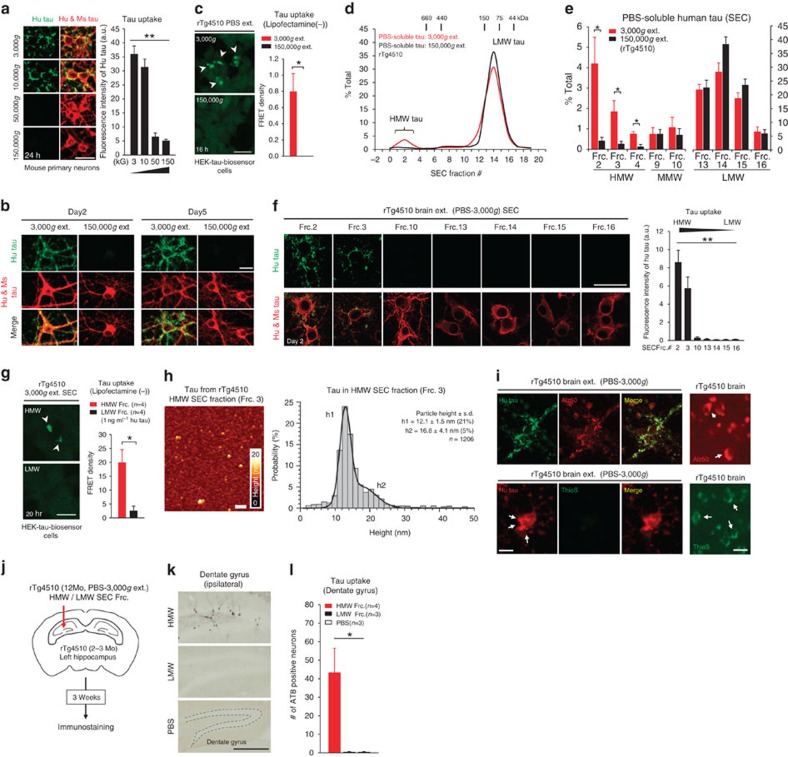
Neuronal uptake of HMW tau from brain extract of rTg4510 tau-transgenic mouse. (**a**) Primary neurons were incubated with PBS-soluble brain extracts (3,000–150,000*g* centrifugation supernatant, 500 ng ml^−1^ human tau) from a 12-month-old rTg4510 mouse. (**a**, left) Immunostaining with human tau-specific antibody (green) and total (human and mouse) tau antibody (red, as a neuronal marker). (**a**, right) Quantification of human tau uptake. (*n*=9–12). One-way ANOVA. (**b**) Neurons were incubated with brain extracts (500 ng ml^−1^ human tau) for 2 and 5 days. (**c**) Tau uptake assay in HEK-tau-biosensor cells. Brain extracts (1 μg protein) were applied to the cells (lipofectamie (−)). (*n*=4). Mann–Whitney *U*-test. (**d**,**e**) SEC of PBS-soluble brain extracts. (**d**) Representative graph of human tau levels (ELISA) in SEC-separated samples. (**e**) Mean human tau levels of HMW (Frc. 2–4), middle molecular weight (Frc. 9–10) and LMW (Frc. 13–16) SEC fractions. (*n*=3). Unpaired *t*-test. (**f**, left) Neurons were incubated with SEC fractions (100 ng ml^−1^ human tau) from 3,000*g* extract and immunostained. (**f**, right) Quantification of human tau uptake. (*n*=3–5). One-way ANOVA. (**g**) Tau uptake assay in HEK-tau-biosensor cells. HMW (Frc. 2)/LMW (Frc. 14) fractions were applied without lipofectamine. (*n*=4). Unpaired *t*-test. (**h**) AFM analysis of HMW tau isolated from rTg4510 brain (10,000*g* total extract, SEC Frc. 3). Full colour range corresponds to a vertical scale of 20 nm. Scale bar, 100 nm. (**h**, right) Size (AFM heights) distribution histogram of HMW tau. (**i**) Human tau taken by neurons was co-stained with Alz50 antibody or ThioS. Brain sections from rTg4510 mouse were used as positive controls for each staining. (**j**–**l**) HMW tau uptake into neurons *in vivo*. (**j**) HMW (Frc. 2–3)/LMW (Frc. 13–14) SEC fractions (rTg4510, PBS-3,000*g*, 100 ng ml^−1^ human tau) or PBS were injected into the left hippocampus of pre-tangle stage rTg4510 mice (2–3 months). (**k**) Three weeks after the injection, the brains were collected and immunostained for tau (AT8). Scale bar, 500 μm. (**l**) Quantification of AT8-positive neurons in the ipsilateral dentate gyrus (Kruskal–Wallis test). Scale bar, 25 μm, except for (**h**) and (**k**). *P<0.05, **P<0.01.

Identification and characterization of tau species taken up by neurons is critical for understanding the mechanism of neuron-to-neuron tau propagation. We first examined the molecular weight of tau species involved in neuronal uptake. We prepared PBS-soluble brain extracts from rTg4510 mice, which overexpress human mutant P301L tau, by centrifugation either at 3,000, 10,000, 50,000 or 150,000*g*, and applied the supernatant to mouse primary cortical neurons. The uptake of tau was assessed by immunofluorescence labelling of intracellular human tau. After 24 h, human tau uptake was observed in neurons treated with 3,000 and 10,000*g* brain extracts, which presumably contained HMW proteins. No uptake occurred from 50,000 and 150,000*g* extracts (Fig. 1a) from which HMW tau was depleted by sedimentation. In neurons treated for longer incubation periods, robust tau uptake was observed from 3,000*g* extract after 2 and 5 days, however, little uptake occurred from 150,000*g* extract even after 5 days of incubation (Fig. 1b). We also confirmed cellular tau uptake from the 3,000*g* extract using fluorescence resonance energy transfer (FRET)-based HEK-tau-biosensor cells^23^ (Fig. 1c). The 3,000*g* brain extracts showed significantly higher seeding activity than 150,000*g* extracts (Supplementary Fig. 1). The seeding activity of 150,000*g* extracts eventually (within 24 h) caught up with that of 3,000*g* extracts (Supplementary Fig. 1b), suggesting that uptake is the key element in the kinetics of tau uptake and aggregation processes.

We then assessed the molecular weight size distribution of tau species contained in each brain extract by SEC. The 3,000*g* brain extract had a small peak of HMW tau species (SEC Frc. 2–4) in addition to a dominant low molecular weight (LMW) tau peak (SEC Frc. 13–16, 50–150 kDa), while the 150,000*g* brain extract from the same rTg4510 mouse brain had only a LMW tau peak and a trace amount of HMW tau species (Fig. 1d,e). The involvement of HMW tau species in neuronal uptake was confirmed by incubating each SEC fraction with primary neurons (Fig. 1f). The most extensive tau uptake was observed for HMW fractions (Frc. 2, 3). Essentially no detectable uptake was observed from the dramatically more abundant LMW fractions, suggesting that HMW tau species were the forms being taken up. Tau uptake assay in HEK-tau-biosensor cells also demonstrated that HMW tau can be taken up by cells more efficiently than LMW tau species (Fig. 1g).

Exposure to 8 M urea reduced the immunoreactivity of the tau oligomer-specific antibody (T22) in the 3,000*g* brain extract (dot blot, Supplementary Fig. 2a,b), and the HMW smear of tau in the HMW SEC fraction largely disappeared after urea incubation (SDS-PAGE, Supplementary Fig. 2c), suggesting the existence of a multimeric tau assembly in this fraction. To further characterize HMW tau, we immunoprecipitated tau from HMW SEC fraction and characterized it by atomic force microscopy (AFM; Fig. 1h). HMW SEC fraction (Frc. 3) contained small globular but no fibrillar tau aggregates (Fig. 1h). The size (particle height) distribution revealed particles of 12.1±1.5 (h1) and 16.8±4.1 (h2) nm (height±s.d., *n*=1,206; Fig. 1h). It remains open if these tau containing particles are made exclusively of tau or contain other constituents such as proteins and lipids.

Human tau species observed within primary neurons 2–5 days after exposure to rTg4510 brain extract were Alz50 positive (Fig. 1i, top) but negative for Thioflavin-S (ThioS) staining (Fig. 1i, bottom), indicating an early stage of pathological conformation of tau that is taken up. Furthermore, tau species taken up by primary neurons co-localized with subcellular organelle markers such as the Golgi apparatus and the lysosomes at day 3 (Supplementary Fig. 3).

Uptake of human tau occurred in a concentration-dependent manner (Supplementary Fig. 4). Applying different human tau concentrations, we found that the minimum concentration of HMW tau from rTg4510 brain extract required for detection of neuronal uptake was 10 ng ml^−1^ (Supplementary Fig. 4), which is lower than the ISF levels of tau in tau-transgenic mice (∼250 ng ml^−1^ (ref. 24)). Tau uptake in primary neurons occurred in both the presence and absence of glial fibrillary acidic protein (GFAP)-labelled astrocytes (Supplementary Fig. 5a).

Importantly, neuronal uptake of HMW tau occurred *in vivo* as well; human tau uptake in neurons was detected in young rTg4510 (pre-tangle stage) (Fig. 1j–l) and WT (Supplementary Fig. 6) mice injected with the HMW SEC fraction of Tg4510 (12 months) brain extract, but not in those injected with the LMW fractions.

### Phosphorylated HMW tau is taken up by neurons

**Figure 2:**
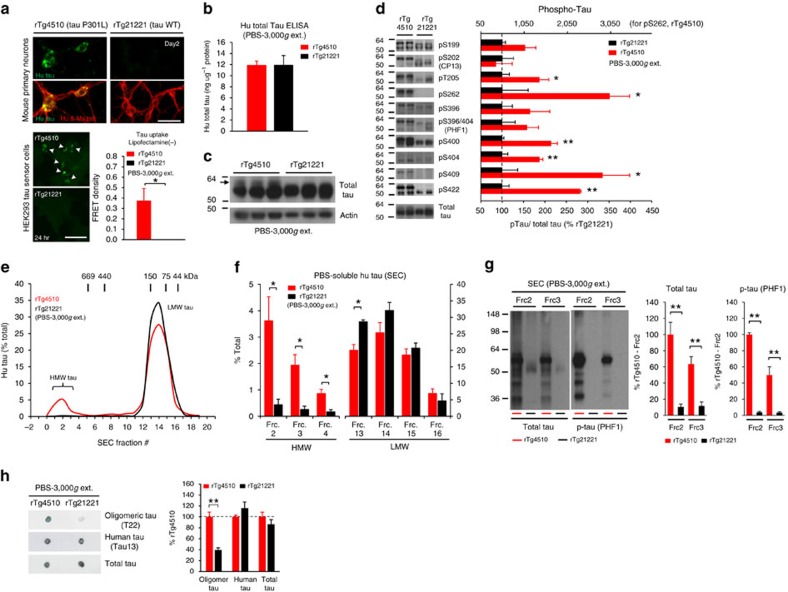
Lack of PBS-soluble phosphorylated HMW tau species is associated with low tau uptake in primary neurons. (**a**, top) Uptake of human tau from brain extracts from rTg4510 and rTg21221 mice by primary neurons (PBS-3,000*g*, 500 ng ml^−1^ human tau). Neurons were immunostained with human tau-specific antibody (green) and total (human and mouse) tau antibody (red). (**a**, bottom) Tau uptake assay in HEK-tau-biosensor cells. Brain extracts (10 μg protein) were applied to the cells (lipofectamie (−)). (*n*=4) Unpaired *t*-test. Scale bar, 50 μm. (**b**) Human tau levels in brain extracts (ELISA). (**c**) Immunoblot analysis of PBS-soluble extracts with total tau antibody (DA9). Up-shifted bands in rTg4510 brain suggest phosphorylation of tau (arrow). (**d**) Brain extracts were immunoblotted with phospho-tau specific antibodies recognizing different epitopes. Representative immunoblot and quantification of phospho-tau levels at each epitope. (*n*=3–4) Unpaired *t*-test. (**e**,**f**) SEC analysis of PBS-soluble tau. (**e**) Representative graph of human tau levels (ELISA) in SEC-separated samples (**f**) Mean human tau levels of HMW (Frc. 2–4) and LMW (Frc. 13–16) SEC fractions. (*n*=3–6) Unpaired *t*-test. (**g**) Immunoblot analysis (SDS-PAGE) of SEC-separated fractions from brain extracts (total tau, DAKO). Quantification of band density is also shown (right graphs) (*n*=4). Unpaired *t*-test. (**h**) Dot blot analysis of PBS-soluble brain extracts with tau oligomer-specific antibody (T22), human tau-specific antibody (Tau13), and total tau antibody. Quantification of dot blot signals is also shown (right) (*n*=4). Unpaired *t*-test. Eleven to thirteen-month-old animals were used. *P<0.05, **P<0.01.

To evaluate the relevance of tau aggregation and phosphorylation for neuronal uptake, we prepared 3,000*g* brain extracts from rTg21221 mice and compared the uptake and biochemical properties with those of rTg4510 homogenate. rTg21221 mice overexpress WT human tau under the same promoter as rTg4510 mice and show phosphorylation but no accumulation of misfolded and aggregated tau species in the brain^20^. Unlike the case for the rTg4510 mice, no uptake was observed in primary neurons from rTg21221 brain extracts at day 2 (Fig. 2a, top). Tau uptake assay in HEK-tau-biosensor cells also demonstrated lack of tau uptake from rTg21221 brain extracts (Fig. 2a, bottom). Human tau and total tau levels in PBS-soluble brain extracts were comparable to those seen in rTg4510 brains (Fig. 2b,c), although an upward shift of the tau band in western blot (Fig. 2c, arrow) suggested a higher degree of tau phosphorylation in rTg4510 brain.

We next compared the degree of tau phosphorylation in rTg4510 and rTg21221 extracts in more detail using 10 different phospho-tau epitope-specific antibodies (Fig. 2d). The PBS-extractable tau species from rTg4510 brain had higher levels of phosphorylation compared with the tau species obtained from rTg21221, especially those associated with some specific phosphorylation sites such as pT205, pS262, pS400, pS404, pS409 and pS422. SEC analysis of the molecular weight distribution of tau demonstrated that rTg21221 brain extracts (PBS-soluble, 3,000*g*) contained primarily LMW species and very low levels of HMW tau species, whereas rTg4510 brain extract showed both HMW and LMW peaks (Fig. 2e,f). The degree of tau uptake into primary neurons correlated significantly with HMW (SEC Frc. 2–4, >669 kDa) tau levels, but not with middle molecular weight (SEC Frc. 9–10, 200–300 kDa) or LMW (SEC Frc. 13–16, 50–150 kDa) tau levels (Supplementary Fig. 7). The differences in HMW tau levels between rTg4510 and rTg21221 brain extracts were also confirmed by western blot (SDS-PAGE) analysis of SEC fractions (Fig. 2g) and semi-denaturing detergent agarose gel electrophoresis (SDD-AGE) blot (Supplementary Fig. 8); HMW tau from rTg4510 brain was highly phosphorylated (Fig. 2g, PHF1). Dot blot analysis demonstrated the presence of oligomeric form of tau in PBS-soluble extracts from rTg4510 brain, although those from rTg21221 brain only had a small amount of tau oligomer assessed with antibody T22 (Fig. 2h). These findings suggest that phosphorylated HMW tau species are taken up by neurons.

### A microfluidic device modelling neuron-to-neuron interactions

**Figure 3:**
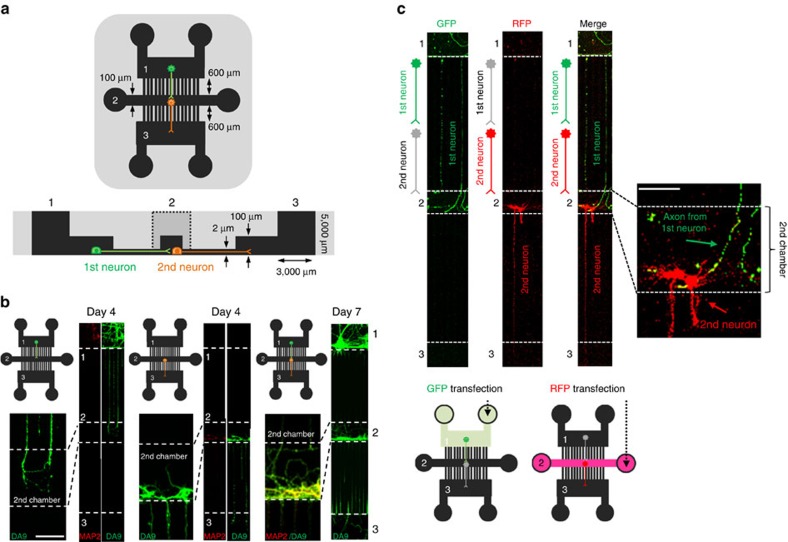
Three-chambered microfluidic device for modelling dual-layered neurons. (**a**) Schematics of a microfluidic device for culturing neurons in three distinct chambers. Mouse primary neurons are plated into the 1st and 2nd chambers (100 μm in thickness) and axon growth is guided through microgrooves (3 μm in thickness, 600 μm in length) connecting each chamber. (**b**, left) Axons from the 1st chamber neuron (green, DA9 as axonal marker) extend into the 2nd chamber within 4 days (neurons were plated only in the 1st chamber). No MAP2-positive dendrites (red) were found in the 2nd chamber, confirming that a 600 μm microgroove is sufficiently long to isolate axon terminals from soma and dendrites. (**b**, middle) Most axons from the 2nd chamber neuron extend into the 3rd chamber (neurons were plated only in the 2nd chamber). (**b**, right) Two sets of neurons were plated into the 1st and 2nd chamber and established synaptic contact in the 2nd chamber. (**c**) Neurons in the 1st and 2nd chambers were transfected with GFP and RFP, respectively. GFP positive axon from the 1st chamber neuron extended into the 2nd chamber, connecting to RFP positive 2nd chamber neuron, which projected its axon into the 3rd chamber. Scale bar, 50 μm.

We then studied the transfer of tau between neurons using a microfluidic neuron culture platform. The design of this platform includes three distinct chambers forming layering synaptic connections between neurons, which are plated on different chambers and arrays of microgrooves, allowing an exclusive axon growth by sizes (Fig. 3a). Two sets of neurons are plated into the 1st and 2nd chambers (Fig. 3a). The axons from the 1st chamber neurons extend into the 2nd chamber within 4 days (Fig. 3b, left), and axons from the 2nd chamber neurons extend to the 3rd chamber (Fig. 3b, middle). The resulting two sets of neurons therefore have ‘in line’ synaptic connections at the 2nd chamber (Fig. 3b, right). We labelled the 1st chamber neurons with green fluorescent protein (GFP) and the 2nd chamber neurons with red fluorescent protein (RFP) using a hydrostatic pressure barrier to fluidically isolate neurons in different chambers^25^. This confirmed that the two neuronal populations were connected to each other in the 2nd chamber (Fig. 3c).

### Neuron-to-neuron transfer of tau in the microfluidic chamber

**Figure 4:**
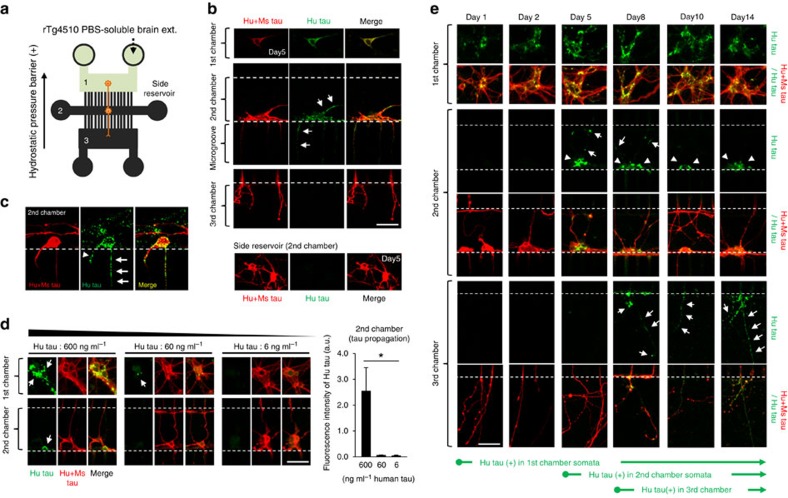
Neuron-to-neuron transfer of rTg4510 mouse brain-derived human tau species in a three-chambered microfluidic device. (**a**) PBS-soluble extract from rTg4510 brain (12-month-old, 500 ng ml^−1^ human tau) was added to the 1st chamber of a 3-chamber microfluidic device. Diffusion of brain extract from the 1st to the 2nd chamber was blocked by a hydrostatic pressure barrier. (**b**) Immunostaining for human tau (green) and total (human and mouse) tau (red) at day 5. Human tau positive neurons were detected in the 2nd chamber (white arrow). Neurons in the side reservoir of the 2nd chamber were negative for human tau staining (bottom). (**c**) A human tau positive axon (arrow) and dendrite (arrow head) extending from the 2nd chamber neuron. (**d**) Concentration dependency of tau uptake and propagation. rTg4510 brain extract (PBS-3,000*g*) was diluted in culture medium to obtain three different concentrations (6, 60 and 600 ng ml^−1^) of human tau and added into the 1st chamber. Neurons were immunostained for human tau and total (human and mouse) tau at day 5. (**d**, right) Quantification of fluorescence intensity of human tau staining in the 2nd chamber. (*n*=4–7). One-way ANOVA. (**e**) Time course of neuron-to-neuron transfer of rTg4510 brain-derived human tau. The rTg4510 brain extract (PBS-3,000*g*, 500 ng ml^−1^ human tau) was added to the 1st chamber and incubated for up to 14 days. Neurons were immunostained at different time points. Human tau positive 2nd chamber neurons (arrow head) and axons from the 1st chamber neuron (arrow) were detected after 5 days of incubation. Human tau positive axons were detected in the 3rd chamber after 8 days (arrow). Scale bar, 50 μm. *P<0.05.

We assessed the propagation properties of rTg4510 brain-derived tau species using the three-chamber microfluidic neuron chamber. PBS-soluble brain extracts from an rTg4510 mouse (3,000*g*, 500 ng ml^−1^ human tau) were added to the 1st chamber (Fig. 4a). To assure that only the 1st chamber neurons were exposed to brain extract, the diffusion-driven transport of various tau species was blocked by convective flow in the opposite direction (hydrostatic pressure barrier). After 5 days of incubation, a human tau-specific immunostain revealed positive immunoreactivity in neurons and in axons of the neurons from the 1st chamber, as well as the soma of the neurons in the 2nd chamber (Fig. 4b), indicating that human tau species taken up by the 1st chamber neurons had been transported through their axons and transferred into the 2nd chamber neurons. Neurons that establish little or no axon-dendrite connection with the 1st chamber neurons (in the side reservoir of the 2nd chamber) remained negative for human tau staining (Fig. 4b, bottom), supporting the idea that axonal input from the 1st chamber is necessary for tau transfer. Human tau was also detected in axons and dendrites extending from the human tau positive 2nd chamber neurons (Fig. 4c), implicating further transport of tau species into the 3rd chamber. Retrograde propagation from the 2nd to the 1st chamber also occurred during the same time course of the experiment (Supplementary Fig. 9), which is consistent with previous research *in vitro*^26^ and *in vivo*^13^,^15^.

The propagation of tau in the microfluidic device was concentration dependent and 500–600 ng ml^−1^ of human tau (in the 1st chamber) was needed to detect propagation to the 2nd chamber neurons over the course of a few days (Fig. 4d,e). Importantly, these concentrations are similar to the ISF tau levels in tau-transgenic mice^24^. Time-course analysis showed early uptake of human tau in the 1st chamber neurons (as early as day 1), propagation to the 2nd chamber neurons after 5 days, and progression to the 2nd chamber neuron axon terminals in the 3rd chamber after 8 days (Fig. 4e). There was no detectable astrocyte contamination in the 2nd chamber of the microfluidic device (Supplementary Fig. 5b), indicating that neuron-to-neuron tau transfer can occur in the absence of astrocytes.

### Persistent transport and lifetime of internalized HMW tau

**Figure 5:**
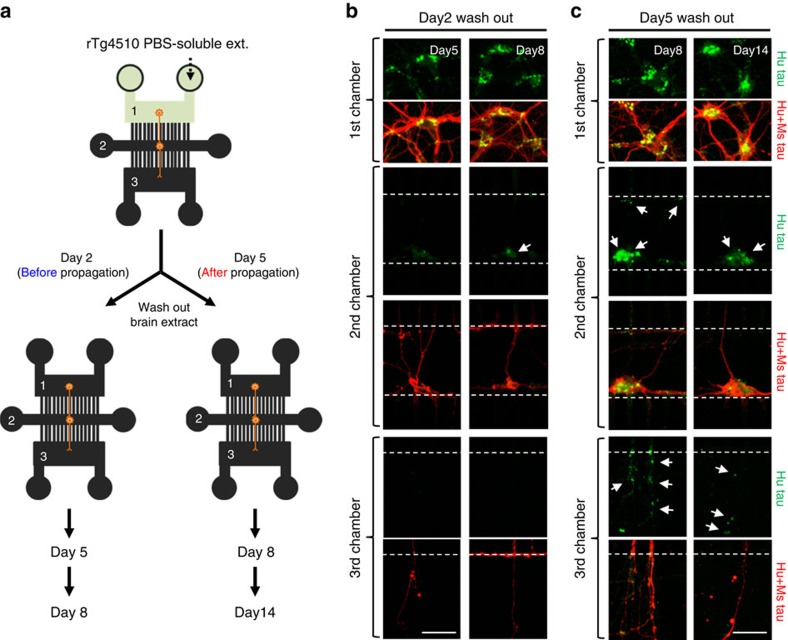
rTg4510 brain-derived human tau was stable and propagated even after removal of brain extract from the chamber. (**a**) rTg4510 brain extract (12-month-old, PBS-3,000*g*) was diluted in culture medium (500 ng ml^−1^ human tau in final concentration) and added to the 1st chamber of 3-chamber microfluidic neuron device. After 2 days (before tau propagation occurs) or 5 days (after tau propagation occurred, but not yet progressed to the 3rd chamber) of incubation, brain extract was washed out from the 1st chamber and replaced with fresh culture medium. (**b**,**c**) Neurons were immunostained for human tau (green) and total (human and mouse) tau (red) at designated time points. (**b**) Human tau positive neuron was detected in the 2nd chamber (day 8, arrow) even after Tg brain extract was washed out from the 1st chamber at day 2. (**c**) Human tau was detected in the 3rd chamber axons (arrow) even after Tg brain extract was washed out from the 1st chamber at day 5. Human tau taken up by the 1st chamber neuron was still detectable at day 14 (9 days after removal of Tg brain extract). Scale bar, 50 μm.

To assess the lifetime of tau in primary neurons after uptake, brain extract from rTg4510 (PBS-3,000*g*, 500 ng ml^−1^ human tau) was added into the 1st chamber of the microfluidic device and excess tau was removed before (at day 2, Fig. 4e) or after (at day 5, Fig. 4e) tau had propagated to the 2nd chamber neurons, and neurons were further cultured for 6 (day 2–8) or 9 (day 5–14) days, respectively (Fig. 5a). Interestingly, human tau positive neurons in the 2nd chamber were detected even after removal of brain extract from the 1st chamber before propagation (at day 8, 6 days after excess tau removal; Fig. 5b). Similarly, human tau positive axons were observed in the 3rd chamber at day 8 (3 days after removal of brain extract from the 1st chamber) (Fig. 5c). These findings indicate that once a certain amount of tau was taken up by the neurons, tau could propagate to the next neuron even after removal of extracellular tau species. Tau species taken up by the 1st chamber neurons or propagated to the 2nd chamber neurons could be detected for up to 6 days (day 2–8, Fig. 5b) or 9 days (day 5–14, Fig. 5c) after washing out the human tau from the medium, indicating a slow degradation of HMW tau species in cultured neurons.

### Uptake of phosphorylated HMW tau derived from AD brains

**Figure 6:**
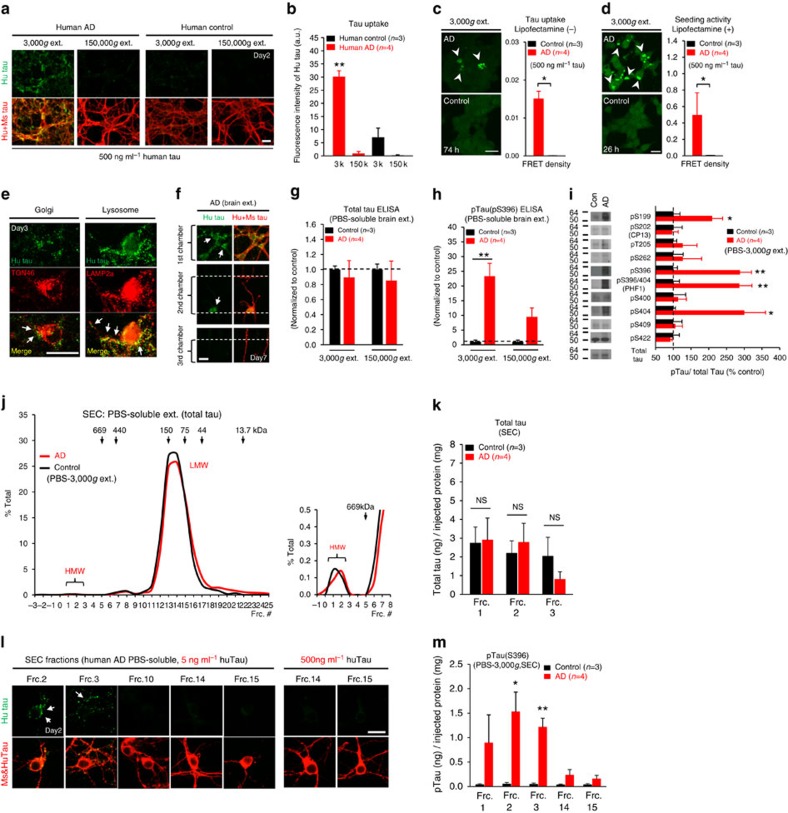
Neuronal uptake of PBS-soluble HMW tau derived from human AD brain. (**a**,**b**) Primary neurons were incubated with AD or control brain extracts (cases were matched for age and postmortem interval (Supplementary Table S1)) and immunostained at day 2 (**a**). (**b**) Quantification of fluorescence intensity of human tau staining. One-way ANOVA and a subsequent Tukey-Kramer test. (**c**,**d**) Tau uptake (**c**) and seeding activity (**d**) assay in HEK-tau-biosensor cells. (Mann–Whitney *U*-test) (**e**) Subcellular localization of human tau taken up by neurons (PBS-3,000*g*, 500 ng ml^−1^ human tau). (**f**) Neuron-to-neuron transfer of tau in a 3-chamber microfluidic device. AD brain extract (PBS-3,000*g*, 500 ng ml^−1^ human tau) was added to the 1st chamber. Human tau positive neurons were detected in both the 1st and 2nd chamber at day 7 (arrow). (**g**,**h**) Quantification of total-tau (**g**) and phospho-tau (**h**) levels in AD and control brain extract (ELISA). Unpaired *t*-test. (**i**) Brain extracts were immunoblotted with phospho-tau specific antibodies recognizing different epitopes. Representative immunoblot and quantification of phospho-tau levels at each epitope. Unpaired *t*-test. (**j**,**k**) SEC analysis of PBS-soluble tau from AD and control brain. (**j**) Representative graph of total tau levels (ELISA) in SEC-separated samples. Small peaks for HMW fractions were detected in both groups (right panel). (**k**) Mean total tau levels of HMW SEC fractions. (**l**) Tau uptake from each SEC fraction (5 or 500 ng ml^−1^ human tau) by primary neurons. (**m**) Phospho-tau levels in each SEC fraction (ELISA). Unpaired *t*-test. Scale bar, 25 μm. *P<0.05, **P<0.01.

We next examined uptake and propagation properties of tau species derived from human AD and control brain tissues. Like rTg4510 brain extract, the PBS-soluble extract from human AD brain contained tau species that could be taken up by mouse primary neurons (Fig. 6a). These tau species again were found only in the 3,000*g* extract (Fig. 6a,b). No uptake was observed from human control brain extracts (Fig. 6a,b). Cellular uptake of AD brain-derived tau (3,000*g* extract) was also confirmed in HEK-tau-biosensor cells (Fig. 6c). The 3,000*g* extracts from AD brain had higher seeding activity than those from control brain (Fig. 6d). The tau species taken up by neurons co-localized with markers for the Golgi apparatus and the lysosomes (Fig. 6e), verifying the internalization and intracellular processing of tau. The tau species from AD brain extracts also propagated between neurons in the three-chamber microfluidic device within 7 days (Fig. 6f).

Total tau levels in PBS-soluble extracts from AD and control brains were similar (Fig. 6g). However, the AD brain extract (3,000*g*) contained significantly higher levels of phosphorylated tau (Fig. 6h,i,m) when compared with the control brain, especially those associated with some specific phosphorylation sites such as pS199, pS396 and pS404 (Fig. 6i). Interestingly, both AD and control brain extracts (PBS-3,000*g*) had comparable total amounts of HMW tau species on SEC analysis (Fig. 6j,k), despite the clear difference in cellular uptake of tau from the AD and control extracts (Fig. 6a–c). The involvement of AD brain-derived HMW tau species in neuronal uptake was confirmed by incubating each SEC fraction with primary neurons (Fig. 6l). Little uptake of the lower molecular weight fractions occurred, even when tau was supplied at 100 times higher concentrations (5 versus 500 ng ml^−1^ human tau in the medium).

We then measured phosphorylation levels of tau in each SEC fraction. The HMW tau species from the AD brain were highly phosphorylated compared with those from control brain (Fig. 6m). Notably, most of the highly phosphorylated tau species from PBS-soluble AD brain extract were detected in the HMW fractions (Fig. 6m). These findings confirmed the presence of phosphorylated HMW tau species in PBS-soluble extracts from AD brain tissue and suggested that these phosphorylated forms may be the forms taken up and propagated by neurons.

### Phosphorylation of tau correlates with neuronal uptake

**Figure 7:**
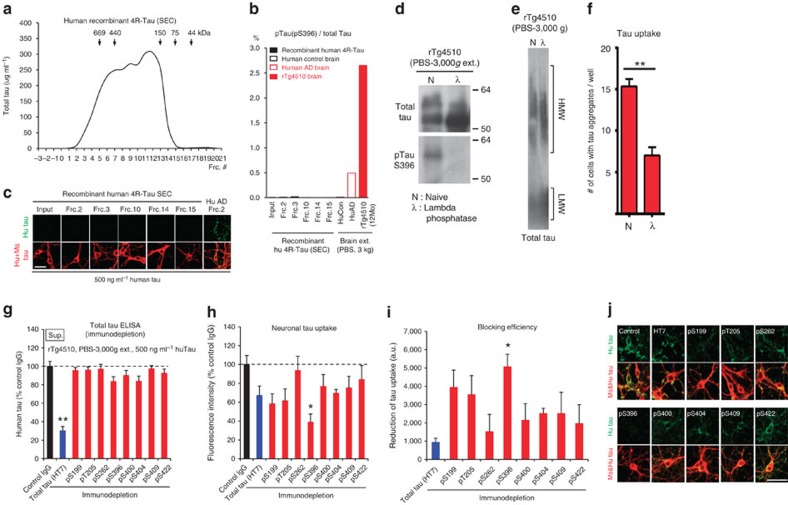
Tau phosphorylation correlates with cellular uptake. (**a**–**c**) Non-phosphorylated HMW tau was not taken up by neurons. (**a**) Tau oligomer mixture solution was prepared from recombinant human tau, followed by SEC and tau ELISA. (**b**) Phospho-tau levels in SEC fractions and brain extracts (pS396 tau ELISA). (**c**) Each SEC fraction was incubated with primary neurons. Neurons were immunostained at day 2. (**d**–**f**) Dephosphorylation reduced tau uptake. (**d**) Immunoblot analysis of total (Tau13)- and phospho-tau (pS396) levels in rTg4510 (12-month-old) brain extracts treated with lambda phosphatase. (**e**) SDD-AGE analysis of brain extracts treated with phosphatase. (**f**) Tau uptake assay. Phosphatase-treated brain extract was applied to HEK-tau-biosensor cells. (*n*=3, **P<0.01), unpaired *t*-test. (**g**–**j**) Immunodepletion of phospho-tau reduced neuronal tau uptake. rTg4510 (12-month-old) brain extracts were immunodepleted with total- or phospho-tau specific antibodies. (*n*=5). (**g**) Total tau levels in tau-immunodepleted samples (ELISA). **P<0.01 versus control IgG. (**h**) Tau uptake in primary neurons (day 2). *P<0.05 versus control IgG. (**i**) Blocking efficiency was defined as the percentage of tau-uptake reduction (versus control IgG) multiplied by tau levels in the immunodepleted brain extracts (% control IgG). *P<0.05 versus total tau (HT7). One-way ANOVA and a subsequent Tukey-Kramer test. (**j**) Representative images of tau uptake in primary neurons. Scale bar, 50 μm.

We tested the idea that tau phosphorylation or, simply, size of tau, was important for cellular uptake by preparing a monomer-dimer-oligomer tau mixture from recombinant human WT full-length tau (441 aa), separated by SEC (Fig. 7a). We then incubated each SEC fraction of this non-phosphorylated tau mixture (Fig. 7b) with mouse primary neurons. No uptake was observed in primary neurons even from HMW tau fractions (Fig. 7c).

We next studied the effect of dephosphorylation of tau on cellular uptake. Phosphatase treatment dephosphorylated tau in rTg4510 brain extract (Fig. 7d) without changing HMW tau levels (Fig. 7e), resulting in a significant reduction of cellular uptake of tau (Fig. 7f). We then assessed tau uptake from phospho-tau-immunodepleted rTg4510 brain extract in primary neurons. Phospho-tau specific antibodies were less efficient at immunodepletion than the total tau antibody (HT7) (Fig. 7g); however, some phospho-tau antibodies (pS199, pT205 and pS396) more efficiently reduced the neuronal tau uptake (Fig. 7h–j) than the total tau antibody, suggesting that they specifically interacted with a species of tau important for uptake. Taken together, these observations indicate that phosphorylation enhances neuronal uptake.

### Brain extracellular tau can be taken up by primary neurons

**Figure 8:**
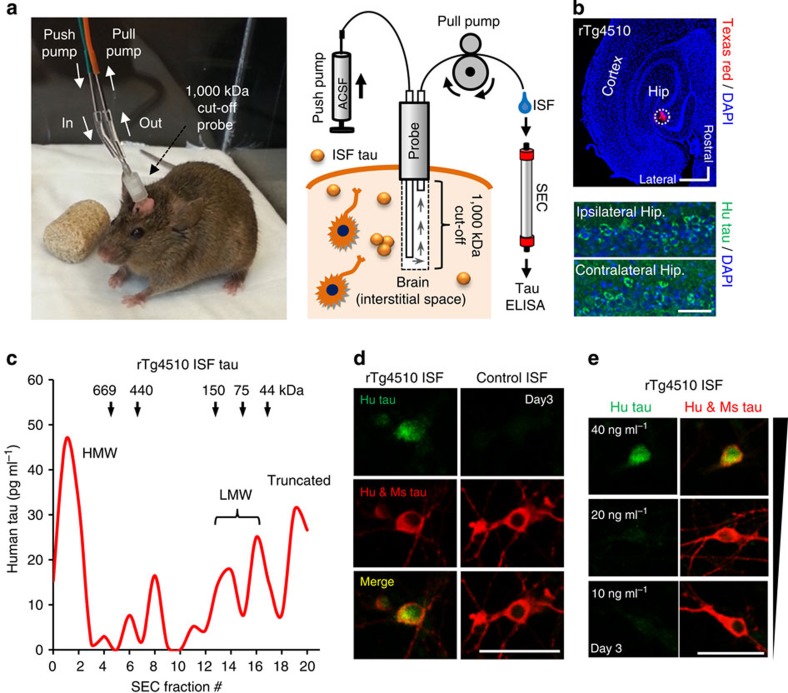
Extracellular tau species from rTg4510 mouse brain can be taken up by primary neurons. (**a**) A large-pore probe *in vivo* microdialysis with push–pull perfusion system. ISF samples were collected from freely moving rTg4510 and control mice (7-month-old) using a 1,000 kDa cutoff probe. (**b**) Representative probe placement. Horizontal brain sections were obtained after ISF collection (24 h after probe insertion) and stained for human tau (green) and DAPI. Dotted line depicts probe location (top). The probe was briefly perfused with Texas red dye (70 kDa, 1 mg ml^−1^) to locate the site of microdialysis. There was no morphological evidence of substantial neuronal loss. There was no apparent difference in the number of human tau positive neurons between ipsilateral (probe-implanted side) and contralateral hippocampal sections (**b**, bottom). Hip, hippocampus. (**c**) Representative graph of human tau levels in SEC-separated ISF sample from rTg4510 mouse. Microdialysate (400 ul) was loaded on SEC column and tau levels in each fraction were measured by ELISA. (**d**) ISF samples were incubated with primary neurons, which were then immunostained for human tau and total (human and mouse) tau. ISF from rTg4510 was diluted to a final concentration of 40 ng ml^−1^ human tau and the same volume of ISF from a control mouse was used for incubation. (**e**) Concentration dependency of ISF tau uptake by primary neurons. rTg4510 brain ISF was diluted in culture medium to obtain three different concentrations (10, 20 and 40 ng ml^−1^) of human tau. Scale bar, 50 μm.

It is known that soluble tau species exist in the cerebrospinal fluid and the ISF in the brain^24^, although limitations of the microdialysis probes used to date would have precluded observation of the critical HMW species described here in the postmortem brain. We therefore employed a unique large pore (1,000 kDa cutoff) probe microdialysis technique with push–pull perfusion system that allows consistent collection of HMW molecules from the brain ISF of awake, freely moving mice^21^,^22^ (Fig. 8a,b). SEC fractionation followed by human tau-specific enzyme-linked immunosorbent assay (ELISA) demonstrated that brain ISF from rTg4510 mouse contained HMW tau species in addition to LMW tau (Fig. 8c). ISF tau from rTg4510 mice was taken up by primary neurons after 3 days of incubation (Fig. 8d), with 40 ng ml^−1^ total human tau being sufficient to detect tau uptake (Fig. 8e). The distribution of tau appeared to be more diffuse in soma compared with tau taken up from brain extracts, which might be due to the relatively low tau levels in ISF or a different size distribution pattern (Fig. 8c). These data show that secreted tau, present in the ISF of awake behaving animals, can be taken up by neurons and therefore might account for the propagation of tau across neural systems observed in transgenic models.

### Effect of tau uptake and aggregation on cell viability

To assess the effect of extracellular tau uptake on cell viability, we performed a cell death assay using ethidium homodimer-1 (EthD-1) staining in HEK-tau-biosensor cells. There was no difference in cell viability between tau-aggregate positive and negative cells for up to 4 days (Supplementary Fig. 10). In addition, we assessed the effect of HMW tau species on neuronal viability using 3-(4,5-dimethylthiazol-2-yl)-2,5-diphenyl tetrazolium bromide (MTT) assay. No acute toxicity was observed in mouse primary neurons treated with HMW tau at 48 h (Supplementary Fig. 11). These results imply that cellular tau uptake and subsequent intracellular aggregation do not cause acute cell death.

## Discussion

Identifying the tau species that can be transferred between neurons is essential for understanding mechanisms by which misfolded tau propagates in AD and other tauopathies. Here we characterized the uptake and propagation properties of tau from various sources: brain extracts and ISF from tau-transgenic mice, brain extracts from postmortem AD patients and recombinant human tau protein. We found that a rare HMW tau species, which accounts for only a small fraction (estimated at <1%) of all soluble tau species in the AD samples, was robustly taken up by neurons, whereas uptake of LMW tau was very inefficient. Findings from the microfluidic neuron culture platform confirmed that this rare species is uniquely capable of propagating between neurons. Furthermore, we demonstrated that tau with similar biochemical characteristics can be identified in the brain ISF of rTg4510 animals obtained while they were awake and behaving, raising the possibility that it is a normal product in the brain. This ISF can also donate tau that can be taken up by neurons in culture. Together, these data imply that (i) a relatively rare, HMW, phosphorylated tau species is released from neurons and found in brain ISF; and (ii) this species can be taken up, axonally transported, secreted and taken up by synaptically connected neurons and thus ‘propagated’.

Uptake of tau from mice expressing aggregating P301L tau (rTg4510) depended on tau molecular weight, correlated with the level of phosphorylation. These findings suggest that oligomerization and pathological phosphorylation increased the uptake efficiency of tau. The HMW tau species had a pathologically misfolded conformation (positive for Alz50 antibody staining) and appeared as non-fibrillar structures in AFM. Previous studies reported that synthetic tau fibrils^26^,^27^,^28^,^29^ or fibrillar tau species extracted from tau-transgenic mouse brain^14^,^15^ could be taken up by neurons and induce filamentous tau pathology *in vitro* and *in vivo*. Our observations indicate instead that soluble, misfolded HMW tau present in the extracellular space likely plays a role in propagation. Preliminary data using extracts from a human G389R tau mutation case with frontotemporal dementia that did not have filamentous tau inclusions nonetheless showed tau uptake into neurons from a PBS-soluble extract, thus supporting the conclusion that soluble tau rather than filamentous aggregates support the propagation phenomenon described here.

Our findings do not entirely exclude the involvement of LMW tau species in uptake and propagation. It may well be possible that the concentration of tau used in this study (500 ng ml^−1^ human tau) was too low for uptake of LMW tau species, or that immunostaining was not sensitive enough to detect LMW tau internalized by neurons. Michel *et al*.^30^ demonstrated that extracellular monomeric tau enters SH-SY5Y neuroblastoma cells at concentration as high as 1 μM (∼55 μg ml^−1^). Notably, consistent with our findings, the same study showed that tau aggregates can be released and then internalized by other cells.

The intracellular accumulation of insoluble tau aggregates has long been considered to be toxic to neurons^31^; however, a recent study reported that insoluble tau aggregates are not sufficient to impair neuronal function^32^. Our findings from the cell viability assay (Supplementary Figs 10 and 11) imply that tau uptake and subsequent intracellular aggregation do not cause acute cell death. Further work will be needed to explore the long-term effect of extracellular and taken-up HMW tau on neuronal function.

Spread of tau pathological lesions from medial temporal lobe towards temporal and other neocortical regions correlates well with the extent of cognitive impairment^5^, and the number of tangles appears to increase in each brain area with increasing duration of disease^3^,^33^. If one mechanism for this spread is trans-synaptic propagation of misfolded tau species, as has been postulated, then understanding the parameters that govern this process is of importance in designing effective strategies to slow progression of cognitive changes in AD. Extracellular tau propagation can be divided into uptake, axonal transport, release and re-uptake phases, with pathobiological processes leading to enhanced or diminished rates at each step. Our data suggest that uptake of unmodified LMW tau at physiological concentrations is not detected during the time course of our experiments, whereas uptake of a relatively rare HMW phosphorylated species is very efficient, occurring within 24 h of exposure. Even *in vivo*, injection of the HMW tau, as opposed to the much more abundant LMW tau species, leads to uptake into neurons both in WT (Supplementary Fig. 6) and pre-tangle stage rTg4510 (Fig. 1j–l) mouse brains. Three lines of data support the idea that phosphorylation is important: uptake efficiency correlates with extent of phosphorylation (Figs 2 and 6); enzymatically dephosphorylating the tau blocks uptake (Fig. 7d–f); and some phospho-tau specific antibodies are able to block uptake despite being relatively ineffective in IPing tau from the rTg4510 brain extract (Fig. 7g–j). In addition, recombinant non-phosphorylated tau, even when prepared as a HMW oligomer, is not taken up efficiently by neurons (Fig. 7a–c).

The HMW tau appears to be quite stable once it is taken up, as it is detectable days after it is washed off (Fig. 5), which might be due to the hyperphosphorylation state of this species^34^,^35^. It undergoes axonal transport, is released, and can be taken up by the next neuron. Thus after initial uptake, at least in these model systems, axonal transport, release into a synapse and trans-synaptic propagation seem to occur relatively rapidly. If these data are applicable to human AD, the implication is that formation, release and uptake of the HMW phosphorylated forms of tau are key factors in determining propagation of tau across neural systems. The amount of HMW phospho-tau species accounted for <1% of the total PBS-soluble tau in AD brain extracts, and <10% even in mutant-tau overexpressing rTg4510 mouse brain extracts, in contrast to the much more abundant LMW tau species. These data suggest that targeting HMW tau species may be an effective way of blocking or slowing the tau propagation cascade in AD. Intervention to deplete these specific extracellular tau species might inhibit tau propagation and hence disease progression in tauopathies.

## Materials and methods

### Animals

Eleven- to thirteen-month-old rTg4510, rTg21221 and control animals were used. The rTg4510 (P301L tau) mouse is a well-characterized model of tauopathy, which overexpresses full-length human four-repeat tau (0N4R) with the P301L frontotemporal dementia mutation^19^. The rTg21221 mouse expresses WTT human tau at levels comparable to rTg4510 mouse and does not show accumulation of tau pathology in the brain^20^. Littermate animals with only the activator CK-tTA transgene, which do not overexpress tau, were used as controls. Both male and female mice were used. All experiments were performed under national (United States National Institutes of Health) and institutional (Massachusetts General Hospital Subcommittee for Research Animal Care and the Institutional Animal Care and Use Committee at Harvard Medical School) guidelines. All animal experiments were approved by the Massachusetts General Hospital and McLaughlin Research Institute Institutional Animal Care and Use Committees.

### Human brain samples

Frozen brain tissues from the frontal cortex of four patients with AD, three non-demented control subjects were obtained from the Massachusetts Alzheimer’s Disease Research Center Brain Bank. The demographic characteristics of the subjects are shown in Supplementary Table 1. All the study subjects or their next of kin gave informed consent for the brain donation, and the Massachusetts General Hospital Institutional Review Board approved the study protocol. All the AD subjects fulfilled the NIA-Reagan criteria for high likelihood of AD. Cortical grey matter was weighed and processed as described in the following section (Brain extraction).

### Brain extraction

Mice were perfused with cold PBS containing protease inhibitors (protease inhibitor mixture; Roche, USA), and the brain was rapidly excised and frozen in liquid nitrogen, then stored at −80 °C before use. Brain tissue was homogenized in five volumes (wt/vol) of cold PBS using a Teflon-glass homogenizer. The homogenate was briefly sonicated (Fisher Scientific Sonic Dismembrator Model 100, output 2, 6 × 1 s) and centrifuged at 3,000*g* for 5 min at 4 °C (3,000*g* extract), 10,000*g* for 15 min at 4 °C (10,000*g* extract), 50,000*g* for 30 min at 4 °C (50,000*g* extract) or 150,000*g* for 30 min at 4 °C (150,000*g* extract). The supernatants were collected and stored at −80 °C before use.

### Primary cortical neuron culture

Primary cortical neurons were prepared from cerebral cortices of embryonic day (E) 14–15 CD1 mouse embryos (Charles River Laboratories) as described previously^36^ with modifications. Cortices were dissected out and mechanically dissociated in Neurobasal (Life Technologies, Inc., USA) medium supplemented with 10% foetal bovine serum, 2 mM Glutamax, 100 U ml^−1^ penicillin and 100 g ml^−1^ streptomycin (plating medium), centrifuged at 150 g for 5 min and resuspended in the same medium. Neurons were plated at a density of 0.6 × 10^5^ viable cells on a Lab-Tek 8-well chambered coverglass (Nalge Nunc) or microfluidic devices (see below for cell density and protocol) previously coated with poly-D-lysine (50 μg ml^−1^, Sigma) overnight. Cultures were maintained at 37 °C with 5% CO_2_ in Neurobasal medium with 2% (vol/vol) B27 nutrient, 2 mM Glutamax, 100 U ml^−1^ penicillin and 100 g ml^−1^ streptomycin (culture medium).

### Tau uptake in primary neurons

Mouse primary neurons (7–8 days *in vitro*) were incubated with PBS-soluble brain extracts (3,000, 10,000, 50,000 or 150,000*g* centrifugation supernatant, or SEC fractions from 3,000*g* extract) from mouse (rTg4510 or rTg21221 animals) or human (control or sporadic AD) brain tissues, microdialysate from rTg4510/control mice, or recombinant tau oligomer mixture solution. Neurons were maintained at 37 °C in 5% CO_2_ in a humidified incubator. Each sample was diluted with culture medium to obtain the designated human tau concentrations (measured by human tau ELISA). Neurons were washed extensively with PBS, fixed and immunostained with human tau-specific antibody (Tau13, #MMS-520R, Covance, 1:2,000) to detect exogenously applied human tau in mouse primary neurons at the designated time point. For most experiments described in this study, we used total (human and mouse) tau antibody (#A0024, DAKO, 1:1,000) as a neuronal marker. Each sample was filtered through a 0.2-μm membrane filter to remove large aggregates and fibrils before incubation.

### Tau uptake and seeding activity assay in HEK293-tau-biosensor cells

Stably expressing CFP-/YFP-TauRD(P301S) HEK293 cells (Holmes *et al*.^23^) were plated at 30,000 cells per well in a 96-well PDL-coated plate. The following day, PBS-soluble brain extracts (3,000*g*) were applied at designated concentrations of human tau or total proteins in a total of 40 μl Opti-MEM (#11058-021, Life technologies) with (for tau seeding assay) or without (for tau uptake assay) Lipofectamine 2000 transfection reagent (#11668019, Life technologies). Cells were fixed with 4% paraformaldehyde (PFA) at designated time points after the extracts were applied and confocal images were obtained via FRET channel (excited with a 458-nm laser and fluorescence was captured with 500–550 nm filter). FRET density, defined as the number of FRET-positive tau aggregates multiplied by the mean fluorescence intensity of FRET-positive tau aggregates and then normalized by the number of cells (4,6-diamidino-2-phenylindole (DAPI) staining), was used for quantification analysis. Each condition was performed at least in triplicate.

### *In vivo* tau uptake assay in WT mice

Stereotactic injections of brain extract were performed as described previously with minor modifications^37^. WT mice (male, 3-months-old, male, C57BL6/J) were injected by using a 30-gauge Hamilton microsyringe in the left frontal cortex (bregma +1.3 mm, 1.5 mm lateral to midline, −1.6 mm relative to bregma) at an infusion rate of 0.2 μl min^−1^. HMW (2.5 μl) (Frc. 2–3) or LMW (Frc. 13–14) SEC fractions (100 or 500 ng ml^−1^ human tau) from rTg4510 brain extract (male, 12-months-old, PBS-soluble, 3,000*g*) were injected. The same volume of PBS was injected as a negative control. Mice were killed 48 h after injection and brain sections from frontal cortex were immunostained with human tau-specific antibody (Tau13, #MMS-520R, Covance, 1:2,000), chicken polyclonal anti-NeuN antibody (#ab134014, Abcam, 1:500), and counterstained with DAPI. Anti-mouse Alexa488 (1:1,000) and CY3-labelled anti-chicken IgG (1:1,000) secondary antibodies were used to detect human tau and NeuN, respectively (see also Immunostaining of brain sections). Images were acquired on an AxioImager Z1 epifluorescence microscope (Carl Zeiss, Oberkochen, Germany). Images were semi-quantitatively evaluated for human tau staining by a rater who was blinded to the experimental conditions, using a score from 0 (no human tau labelling on NeuN positive neurons) to 4 (maximum human tau labelling on NeuN positive neurons).

### *In vivo* tau uptake assay in young rTg4510 mice

Hippocampal injections of brain extract were performed as described previously with minor modifications^13^. rTg4510 mice (male, 2–3-months-old, pre-tangle stage) were injected by using a 30-gauge Hamilton microsyringe in the left hippocampus (bregma −2.5 mm, 2.0 mm lateral to midline, −1.8 mm relative to bregma) at an infusion rate of 0.2 μl min^−1^. HMW (2.5 μl) (Frc. 2–3) or LMW (Frc. 13–14) SEC fractions (100 ng ml^−1^ human tau) from rTg4510 brain extract (male, 12-months-old, PBS-soluble, 3,000*g*) were injected. The same volume of PBS was injected as a negative control. Mice were killed three weeks after injection and serial coronal brain sections (40 μm) were taken though the entire brain. Sections were incubated with 0.3% hydrogen peroxide for 10 min at room temperature (R.T.), blocked in 3% milk in Tris buffered saline (TBS) with 0.25% Triton X-100, and incubated with biotinylated AT8 antibody (ThermoScientific, MN1020B, 1:1,000) in 3% milk in TBS with 0.25% Triton X-100 overnight at 4 °C. After washing in TBS, sections were developed with nickel-enhanced DAB substrate using the VECTASTAIN Elite ABC Kit (Vector Laboratories). Every seventh section was stained. Images were obtained using an Olympus BX51 microscope mounted with a DP 70 Olympus digital camera. The number of AT8-positive neurons was manually counted by a blinded investigator (seven sections for each mouse).

### Atomic force microscopy

Immunoprecipitation isolation of tau from rTg4510 brain extract for AFM analysis was performed as described previously with minor modifications^38^. Briefly, tosylactivated magnetic Dynabeads (#14203, Life Technologies) were coated with human tau-specific Tau13 antibody. Beads were washed (0.2 M Tris, 0.1% bovine serum albumin, pH 8.5) and incubated with HMW SEC fraction (Frc. 3 from 10,000*g* extract, rTg4510) sample for 1 h at R.T. Beads were washed three times with PBS and eluted using 0.1 M glycine, pH 2.8, and the pH of each eluted fraction was immediately adjusted using 1 M Tris pH 8.0. For AFM imaging, isolated tau fractions were adsorbed onto freshly cleaved muscovite mica and imaged using oscillation mode AFM (Nanoscope III, Di-Veeco, Santa Barbara, CA) and Si_3_N_4_ cantilevers (NPS series, Di-Veeco) in PBS, as described previously^39^. For size (AFM heights) distribution histogram of HMW tau oligomers (SEC Frc. 3), 1,206 particles from seven randomly picked images (1.5 × 1.5 μm) were analysed (Fig. 1h).

### *In vivo* microdialysis

*In vivo* microdialysis sampling of brain ISF tau was performed as described previously^21^,^22^. The microdialysis probe had a 4 mm shaft with a 3.0 mm, 1,000 kDa molecular weight cutoff polyethylene membrane (PEP-4-03, Eicom, Japan). This probe contains a ventilation hole near the top which serves to produce a reservoir of fluid within the probe that is open to the atmosphere. This structure minimizes pressure which would otherwise cause a net flow of perfusate out through the large-pore membrane. Before use, the probe was conditioned by briefly dipping it in ethanol, and then washed with an artificial cerebrospinal fluid perfusion buffer (in mm: 122 NaCl, 1.3 CaCl_2_, 1.2 MgCl_2_, 3.0 KH_2_PO_4_ and 25.0 NaHCO_3_) that was filtered through a 0.2 μm pore-size membrane. The preconditioned probe’s outlet and inlet were connected to a peristaltic pump (ERP-10, Eicom) and a microsyringe pump (ESP-32, Eicom), respectively, using fluorinated ethylene propylene (FEP) tubing (φ 250 μm inner diameter).

Probe implantation was performed as previously described^21^, with slight modifications. Briefly, the animals were anesthetized with isoflurane, while a guide cannula (PEG-4, Eicom) was stereotactically implanted in the hippocampus (bregma −3.1 mm, −2.5 mm lateral to midline, −1.0 mm ventral to dura).

Three or four days after the implantation of the guide cannula, the mice were placed in a standard microdialysis cage and a probe was inserted through the guide. After insertion of the probe, to obtain stable recordings, the probe and connecting tubes were perfused with artificial cerebrospinal fluid for 180 min at a flow rate of 10 μl min^−1^ before sample collection. Samples were collected at a flow rate of 0.5 μl min^−1^.

### Tau ELISA

The concentrations of human tau in the samples (brain extracts, brain ISF samples, and recombinant human tau solution, and SEC-separated samples) were determined by Tau (total) Human ELISA kit (#KHB0041, Life Technologies) and Tau [pS396] Human ELISA kit (#KHB7031, Life Technologies), according to the manufacturer’s instructions.

### Immunoblot analysis

Brain extracts were electrophoresed on Novex Tris-Glycine gels (Life Technologies, Grand Island, NY, USA) in Tris-Glycine SDS running buffer for SDS-PAGE (Life Technologies). Gels were transferred to PVDF membranes, and membranes were blocked for 60 min at R.T. in 5% (wt/vol) bovine serum albumin (BSA)/TBS-Tween (TBS-T), and then probed with primary antibodies overnight at 4 °C in 2% (wt/vol) BSA/TBS-T. The following primary antibodies were used: mouse monoclonal antibody DA9 (total tau (aa112–129), courtesy of Peter Davies, 1:5,000), mouse monoclonal antibody PHF1 (pS396/pS404 tau, courtesy of Peter Davies, 1:5,000), mouse monoclonal antibody CP13 (pS202 tau, courtesy of Peter Davies, 1:1,000), rabbit polyclonal anti-phospho tau antibodies (pS199 (#44734G), pT205 (#44738G), pS262 (#44750G), pS396 (#44752), pS400 (#44754G), pS404 (#44758G), pS409 (#44760G) and pS422 (#44764G)) from Life Technologies (1:2,000 dilution for these antibodies), and mouse monoclonal anti-actin antibody (#A4700, Sigma-Aldrich, 1:2,500). After washing three times in PBS-T, blots were incubated with HRP-conjugated goat anti-mouse (#172-1011, Bio-Rad) or anti-rabbit (#172-1019, Bio-Rad) IgG secondary antibodies (1:2,000 dilution) for 1 h at R.T. Immunoreactive proteins were developed using an ECL kit (Western Lightning, PerkinElmer, USA) and detected on Hyperfilm ECL (GE healthcare, USA). Protein/lane (15 μg) were loaded, unless indicated otherwise. Scanned images were analysed using Image J (National Institutes of Health). Full length versions of western blots are shown in Supplementary Fig. 12.

### Dot blot analysis

For dot blot, brain extracts (0.75 μg protein in 1.5 μl) were spotted directly onto nitrocellulose membranes (#88018, Thermo Scientific). Membranes were blocked for 60 min at R.T. in 5% (wt/vol) BSA/TBS-T, and then probed with primary antibodies for 60 min at R.T. in 2% (wt/vol) BSA/TBS-T. The following primary antibodies were used: rabbit polyclonal tau oligomer-specific antibody T22 (#ABN454, Millipore, 1:1,000)^40^, mouse monoclonal antibody Tau13 (#MMS-520R, Covance, 1:2,000), rabbit polyclonal anti-total tau antibody (#ab64193, Abcam, 1:1,000). After washing three times in PBS-T, blots were incubated with HRP-conjugated goat anti-mouse (#172-1011, Bio-Rad) or anti-rabbit (#172-1019, Bio-Rad) IgG secondary antibodies (1:2,000 dilution) for 60 min at R.T. Immunoreactive proteins were developed using an ECL kit (Western Lightning, PerkinElmer, USA) and detected on Hyperfilm ECL (GE healthcare, USA).

### Urea/SDS treatment

The rTg4510 brain extracts (12-months-old, PBS-3,000*g*) were incubated with 8 M urea or 10% SDS (1.0 μg μl^−1^ total protein in 8 M urea or 10% SDS) for 24 h at 37 °C before application to the membrane for dot blot analysis. The SEC HMW fractions (Frc. 2) from the rTg4510 brain extracts (12-months-old, PBS-3,000*g*) were incubated with 8 M urea for 24 h at 37 °C and analysed by SDS-PAGE (non-reducing condition) using total tau antibody (#A0024, DAKO, 1:1,000).

### Semi-denaturing detergent agarose gel electrophoresis

SDD-AGE was carried out as previously described^13^ with minor modifications. Brain extract was thawed on ice. A 1.5% agarose gel was prepared by dissolving agarose in buffer G (20 mM Tris-Base, 200 mM glycine) and then adding 0.02% SDS. A total of 50 μg (for Tg4510 and Tg21221 brain extracts (Supplementary Fig. 8)) or 25 μg (for lambda phosphatase experiment (Fig. 7e)) of brain extract protein was incubated with 0.02% SDS sample buffer for a total of 7 min at R.T. just before loading. The SDD-AGE was run in Laemmli buffer (Buffer G with 0.1% SDS) at 30 V for 14 h or until the dye front reached the end of the gel. Protein was then transferred via capillary action to Immoblin P (Millipore) membrane at 4 °C for 16–24 h. Membranes were blocked in 5% non-fat dry milk/TBS-T for 1 h and then probed for total tau using rabbit polyclonal anti-tau antibody (#ab64193, Abcam, 1:4,000) overnight at 4 °C. Membranes were washed three times with TBS-T, probed with goat anti-rabbit IgG-HRP (#172-1019, Bio-Rad, 1:2,000) for 1.5 h at R.T., and washed three times with TBS-T. Membranes were developed using ECL kit (Western Lightning, PerkinElmer, USA) and detected on Hyperfilm ECL (GE healthcare, USA).

### Immunodepletion

Immunodepletion of tau from rTg4510 brain extracts was performed using Dynabeads Protein G Immunoprecipitation Kit (#10007D, Life Technologies) according to the manufacturer’s instructions with minor modifications. A 0.75 mg of Dynabeads Protein G was incubated with 1 μg of anti-tau antibodies (anti-phospho tau antibodies (see Immunoblot analysis section), total tau antibody (HT7, #MN1000, Thermo Scientific), and control IgG) for 10 min with rotation at R.T. After washing with 200 μl of washing buffer, the Dynabeads-antibody complex was incubated with 300 μl of rTg4510 brain extracts (12-months-old, PBS-3,000*g*, 500 ng ml^−1^ human tau) for 10 min with rotation at R.T. Dynabeads-antibody–antigen complex was isolated using a magnetic holder and the supernatant was collected for tau uptake assay and ELISA measurement. After washing three times with 100 μl of washing buffer, Dynabeads-antibody–antigen complex was resuspended in 20 μl of elution buffer and incubated for 2 min at R.T. Dynabeads-antibody complex was isolated using a magnetic holder and supernatant (immunoplecipitated tau) was collected for tau ELISA measurement.

### Lambda phosphatase treatment

In all, 25 μg protein of brain extract (12-month-old rTg4510, PBS-3,000*g*) was incubated for 1 h at 30 °C with 400 units of Lambda Protein Phosphatase (NEB) supplemented with 1X NEBuffer for protein metalophosphatase and 1 mM MnCl_2_, immediately followed by 1 h at 65 °C to inactivate the Lambda Phosphatase enzymatic activity.

### Size-exclusion chromatography

Brain PBS-soluble extracts, ISF microdialysate, oligomer tau (recombinant hTau-441) mixture solution were separated by SEC on single Superdex200 10/300GL columns (#17-5175-01, GE Healthcare) in phosphate buffered saline (#P3813, Sigma-Aldrich, filtered through a 0.2-μm membrane filter), at a flow rate of 0.5 ml min^−1^, with an AKTA purifier 10 (GE Healthcare). Each brain extract was diluted with PBS to contain 6,000 ng of human tau in a final volume of 350 μl, which was filtered through a 0.2-μm membrane filter and then loaded onto an SEC column. The individual fractions separated by SEC were analysed by ELISA (Tau (total) Human ELISA kit, diluted 1:50 in kit buffer). For the ISF sample, 400 μl of microdialysate from rTg4510 mice was loaded onto the column after filtration through a 0.2-μm membrane, and SEC fractions were measured by human tau ELISA. For the oligomer tau mixture solution, 500 μl of sample (hTau-441, 3.35 mg ml^−1^ with 2 mM dithiothreitol (DTT), filtered through a 0.2-μm membrane filter) was loaded onto column and each SEC fraction was diluted 1:200,000 in kit buffer for human tau ELISA.

### Immunocytochemistry

Primary neurons were washed extensively with PBS (three times) and fixed with 4% PFA for 15 min. Neurons were washed with PBS, permeabilized with 0.2% Triton X-100 in PBS for 15 min (R.T.), blocked with 5% normal goat serum (NGS) in PBS-T (R.T.), and then incubated with the primary antibodies overnight at 4 °C in 2% NGS/PBS-T. The following primary antibodies were used to detect tau: mouse monoclonal antibody Tau13 (specific for human tau (aa20-35), #MMS-520R, Covance, 1:2,000), rabbit polyclonal anti-total tau antibody (recognizes both human and mouse tau, #A0024, DAKO, 1:1,000), mouse monoclonal antibody Alz50 (the conformation-specific antibody, courtesy of Peter Davies, Albert Einstein College of Medicine; 1:100). Goat anti-mouse Alexa488 and anti-rabbit Alexa555 secondary antibodies (Life Technologies, 1:1,000) were applied in 2% NGS in PBS-T for 1 h at R.T. CY3-labelled anti-mouse IgM secondary antibody (Invitrogen, 1:200) was used to detect Alz50. After washing in PBS, coverslips were mounted with aqueous mounting medium (Vectashield). For co-staining with ThioS, neurons were first immunostained with rabbit polyclonal antibody TAUY9 (specific for human tau (aa12–27), #BML-TA3119-0025, Enzo Life Sciences, 1:200) and goat anti-rabbit Alexa555 secondary antibody (Invitrogen, 1:200), and then incubated with 0.025% (wt/vol) ThioS in 50% ethanol for 8 min. ThioS was differentiated in 80% ethanol for 30 s. Neurons were washed with water for 3 min, and coverslips were mounted using mounting medium (Vectashield). For co-staining with microtubule-associated protein 2 (MAP2) and GFAP, chicken polyclonal anti-MAP2 antibody (#ab5392, Abcam, 1:1,000) and rabbit polyclonal anti-GFAP antibody (#ab7260, Abcam, 1:1,000) were used for primary antibodies, and CY3-labelled anti-chicken IgG (1:1,000) and anti-rabbit Alexa350 (1:500) secondary antibodies were used to detect MAP2 and GFAP, respectively.

The subcellular localization of human tau was studied by co-staining with the following primary antibodies: rabbit polyclonal anti-TGN46 antibody (the Golgi apparatus marker, #ab16059, Abcam, 1:200), rabbit polyclonal anti-GRP94 antibody (the endoplasmic reticulum marker, #ab3670, Abcam, 1:100), rabbit polyclonal anti-LAMP2a antibody (the lysosome marker, #ab18528, Abcam, 1:200), rabbit polyclonal anti-Rab5 antibody (the endosome marker, #ab13253, Abcam, 1:200), rabbit polyclonal anti-catalase antibody (the peroxisome marker, #ab1877, Abcam, 1:200). Goat anti-rabbit Alexa555 secondary antibody (Invitrogen, 1:200) was used to detect subcellular markers. Images were acquired using confocal microscope (Zeiss Axiovert 200 inverted microscope, Carl Zeiss).

### Immunostaining of brain sections

Mice were killed by CO_2_ asphyxiation and perfused with PBS. Brains were fixed in 4% PFA for 2 days at 4 °C, incubated for 2 days in 30% sucrose in PBS, and then 40-μm-thick sections were cut on a freezing sliding microtome. Sections were permeabilized with 0.2% Triton X-100 in PBS, blocked in 5% NGS in PBS, and incubated in primary antibody (mouse monoclonal antibody Alz50 (courtesy of Peter Davies, 1:100)) in 2% NGS in PBS overnight at 4 °C. CY3-labelled anti-mouse IgM secondary antibody (Invitrogen, 1:200) was applied in 2% NGS in PBS for 1 h (R.T.). After washing in PBS, brain slices were mounted on microscope slides, and coverslips were mounted using DAPI containing mounting medium (Vectashield). NFTs were stained with 0.025% ThioS in 50% ethanol for 8 min. ThioS was differentiated in 80% ethanol for 30 s. Sections were washed with water for 3 min, and coverslips were mounted using mounting medium.

### Preparation of tau oligomer mixture solution

Human full-length WT tau (2N4R, 441 aa) was expressed in *Escherichia coli* BL21 DE3 using tau/pET29b plasmid (Addgene). Expression was induced at OD=0.6 by adding 1 mM IPTG for 3.5 h at 37 °C. Tau purification was performed by heat treatment and FPLC Mono S chromatography (Amersham Biosciences) as described previously^41^. Cells of 300 ml culture were boiled in 3 ml buffer solution (50 mM MES, pH 6.8, 500 mM NaCl, 1 mM MgCl_2_, 5 mM DTT) for 20 min. Whole cell lysate was ultracentrifuged at 125,000*g* for 45 min, and supernatant was dialysed (molecular weight cutoff 20 kDa) against 20 mM MES, pH 6.8, 50 mM NaCl, 2 mM DTT. Protein and tau content was determined by BCA assay kit (Pierce), SEC and western blot. Tau oligomer mixture solution was prepared by incubating recombinant human tau (3.35 mg ml^−1^) with 2 mM DTT for 2 days at 37 °C, followed by SEC separation and ELISA measurement of tau (Fig. 7a).

### Microfluidic three-chamber devices

We designed a neuron-layering microfluidic platform composed of three distinct chambers connected through microgroove arrays (3 × 8 × 600 μm in height, width and length) using standard soft lithographic techniques^42^. The length of the microgrooves was such that no MAP2-positive dendrites entered the adjacent chambers (Fig. 3b), in agreement with earlier observations from Taylor *et al*.^25^ who reported that a 450-μm microgrooves are sufficiently long to isolate axon terminals from soma and dendrites. The platform was punched on two side reservoirs of each chamber and bonded to a poly-D-lysine (50 μg ml^−1^, Sigma) coated glass-bottom dish (#P50G-1.5-30-F, MatTek Corporation) to enhance neuronal adhesion.

First, primary cortical neurons isolated from E15 mouse embryos were plated into the 1st chamber at an approximate density of 0.6 × 10^5^ viable cells (in 10 μl plating medium) per device. After 15 min (most neurons in the 1st chamber adhered to the bottom of dish during this period), 0.3 × 10^5^ viable cells in 2 μl plating medium were loaded into the 2nd chamber via one of the side reservoirs. Microfluidic devices were set at a tilt (∼80° angle) in an incubator (37 °C in 5% CO_2_) immediately after neurons were plated into the 2nd chamber, so that the neurons would settle down to surfaces close to the third chamber by gravity. Therefore, most neurons in the 2nd chamber were plated in a line along a sidewall of the 2nd chamber, which had microgrooves connecting to the 3rd chamber. We empirically established this protocol to allow most of the axons of neurons in the 2nd chamber to extend into the 3rd chamber (Fig. 3b). After 3 h, the devices were set in a normal position without tilting and maintained at 37 °C in 5% CO_2_ in culture medium. The medium was changed every 4–5 days.

For tau uptake and the propagation assay, PBS-soluble extracts from rTg4510 and AD brain tissue were added to the 1st chamber (10 μ in total) on 7 or 8 days *in vitro*. The 2nd and 3rd chambers were filled with 40 μl of media (20 μl in each reservoir). The volume difference between the chambers resulted in continuous concevtion (‘hydrostatic pressure barrier’; 10 μl in the 1st chamber and 40 μl in the 2nd and 3rd chambers), which prevented diffusion of brain extract from the 1st chamber into other chambers. After incubation for the designated periods, neurons were washed, fixed and immunostained as described above (see Immunocytochemistry section).

Transfections of GFP and RFP (Fig. 3c) were carried out inside microfluidic devices as follows: initially, the 1st chamber neurons (7 DIV) were transfected with GFP (0.04 μg DNA+0.1 μl of Lipofectamine 2000 (#11668-019, Life Technologies)) in 10 μl of NeuroBasal for 3 h. Diffusion of DNA from the 1st chamber to the 2nd chamber was prevented by a hydrostatic pressure barrier, as described above. After washing the DNA (GFP)-containing medium from the 1st chamber (washed three times with NeuroBasal), the 2nd chamber neurons were transfected with RFP (0.04 μg DNA+0.1 μl of Lipofectamine 2000 in 10 μl NeuroBasal) for 3 h. Diffusion of DNA from the 2nd chamber to the 1st and 3rd chambers was prevented by the hydrostatic pressure barrier (40 μl media in the 1st and 3rd chambers). After washing the DNA (RFP)-containing medium from the 2nd chamber (washed three times with NeuroBasal), devices were maintained at 37 °C in 5% CO_2_ in culture medium. Expressions of GFP and RFP were examined 2 days later.

Neurons in the microfluidic device were examined using a Zeiss Axiovert 200 inverted microscope (Carl Zeiss) equipped with a Zeiss LSM 510 META (Zeiss, Jena, Germany) confocal scanhead using 488- and 543-nm lasers. All images were acquired using a 25 × APO-Plan Neoflu lens or 63 × 1.2 NA C-APO-Plan Neoflu lens (Carl Zeiss).

### EthD-1 staining

Cell viability assay with EthD-1 staining (#L-3224, Life Technologies) was performed according to the manufacturer’s instructions with minor modifications. Cells were washed twice with PBS and incubated with EthD-1 (4 μM in PBS) and Hoechst 33342 (#H3570, Life Technologies, 1 μg ml^−1^ in PBS) for 20 min at 37 °C in 5% CO_2_ in a humidified incubator. Images were acquired using confocal microscope (Zeiss Axiovert 200 inverted microscope, Carl Zeiss).

### MTT assay

Neuronal viability was assessed using a MTT assay kit (TACS MTT Cell Proliferation Assays, #4890-25-K, Trevigen) according to the manufacturer’s instructions with minor modifications. Mouse primary neurons were incubated with HMW or LMW SEC fractions from rTg4510 brain extracts (12-months-old, PBS-3,000*g*, 10 ng ml^−1^ human tau) in 96-well plate (Corning, #3603) (2.5 × 10^4^ cells/well in 100 μl) for 48 h. MTT (10 μl) was added to each well at 48 h and neurons were incubated for 2 h in an incubator (37 °C in 5% CO_2_). When purple precipitate is visible under the microscope, 100 μl of detergent reagent was added to each well and incubated for 4 h at R.T. The absorbance of each well was measured at 600 nm in a microplate reader (Wallac Victor 1420 Multilabel Counter, Perkin Elmer).

### Statistical analysis

All data were expressed as mean±s.e.m. Two-group comparisons were performed by unpaired *t*-test, unless stated otherwise. Comparison among three or more groups was performed by analysis of variance and Tukey-Kramer multiple range test, unless stated otherwise. Correlations were analysed with Pearson correlation analysis. *P* values <0.05 were considered significant.

## Additional information

**How to cite this article:** Takeda, S. *et al*. Neuronal uptake and propagation of a rare phosphorylated high-molecular-weight tau derived from Alzheimer’s disease brain. *Nat. Commun.* 6:8490 doi: 10.1038/ncomms9490 (2015).

## Supplementary Material

Supplementary Information Supplementary Figures 1-12 and Supplementary Table 1
