# Supplementary material for: Neuronal uptake and propagation of a rare phosphorylated high-molecular-weight tau derived from Alzheimer's disease brain
Source: Nat Commun. 2015 Oct 13;6:8490. doi: 10.1038/ncomms9490 (PMC4608380; doi:10.1038/ncomms9490)
Supplement: Supplementary Information — Supplementary Figures 1-12 and Supplementary Table 1 [file ncomms9490-s1.pdf]

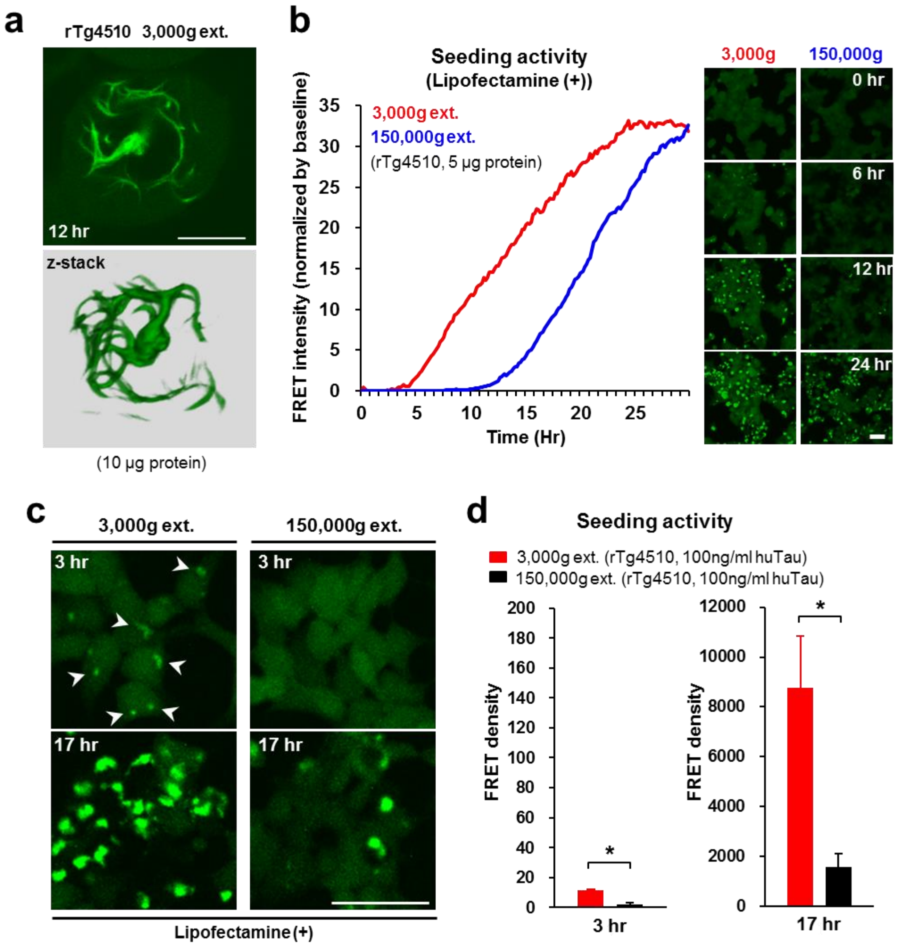

Supplementary Figure 1 Tau seeding activity assay in HEK-tau-biosensor cells. (a) Representative image of intracellular tau aggregate induced by rTg4510 brain extract (PBS-soluble, 3,000g, 10 µg protein). Single confocal (top) and z-stack (3D, bottom) images were taken at 12 hours. Scale bar: 10 µm. (b) Time-course of tau seeding. PBS-soluble 3,000g or 150,000g brain extracts from rTg4510 mice were applied to HEK-tau-biosensor cells with lipofectamine (1%). Time-lapse confocal images (FRET channel; ex. 458nm, em. 500-550nm) were taken every 10 min and fluorescence intensity of the FRET images was measured. (b, left) Representative graph of FRET intensity. (b, right) Confocal images (FRET channel) at 0, 6, 12, and 24 hour time points are shown. 3,000g brain extracts have higher seeding activity than 150,000g extracts. Scale bar: 50 µm. (c, d) Seeding activities of 3,000g and 150,000g brain extracts (*n* = 4 / group) were compared at 3 and 17 hour time points. (c) Representative confocal images of tau aggregates. 3,000g brain extract induced intracellular tau aggregation as early as 3 hours (arrow head). Scale bar: 50 µm. (d) Quantification of FRET density. (*n* = 4 / group) *$P < 0.05$. 12-month-old rTg4510 mice were used.

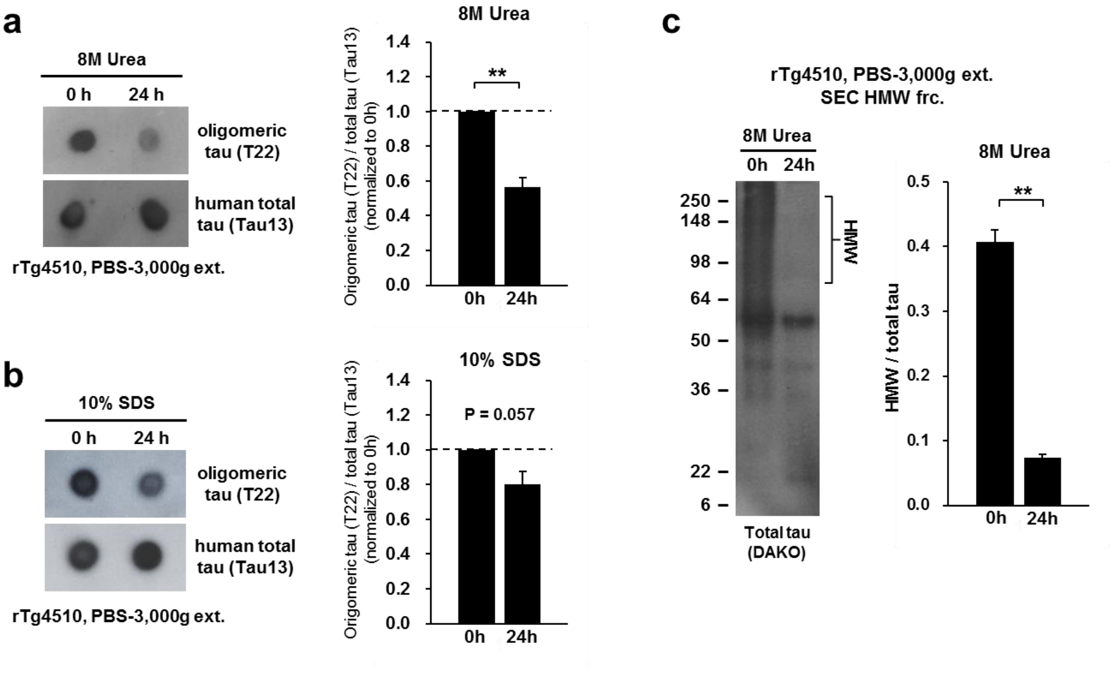

Supplementary Figure 2 Dot blot and SDS-PAGE analysis of HMW tau from rTg4501 brain extracts. (a, b) PBS-3,000g brain extracts from rTg4510 (12 months old) were incubated with 8 M urea (a) or 10% SDS (b) for 24 hours at 37°C and analyzed by dot blot using tau oligomer-specific (T22) and total tau (Tau13) antibodies. Representative images of dot blot (left) and quantification of immunoreactivities of each antibody (right) are shown. Immunoreactivity of the tau oligomer-specific antibody (T22) significantly decreased after exposure to 8 M urea. (n = 5-7) $^{**}P < 0.01$ (paired t-test). (c) SDS-PAGE analysis of the SEC HMW fraction (Frc.2) from rTg4510 brain extracts (PBS-3,000g). The SEC HMW fraction was incubated with 8 M urea for 24 hours at 37°C and analyzed by SDS-PAGE using total tau (DAKO) antibody. Representative blot (left) and quantification (right) are shown. (n = 3) $^{**}P < 0.01$ (paired t-test). The HMW smear disappeared after exposure to 8 M urea, suggesting the existence of a multimeric tau assembly in the HMW fraction.

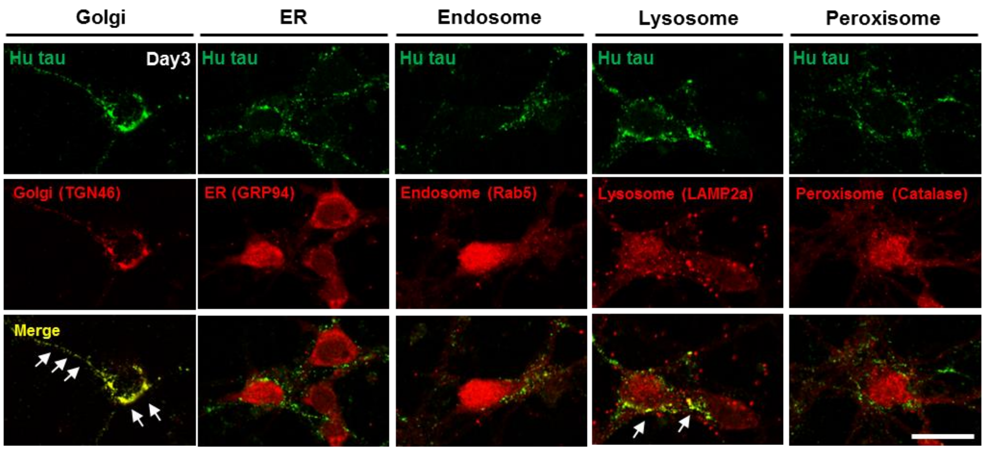

Supplementary Figure 3 Subcellular localization of tau taken up by neurons. Mouse primary neurons were incubated with PBS-soluble brain extracts (3,000g, 500 ng/ml human tau) from a 12-month-old rTg4510 mouse and immunostained with human tau specific antibody (Tau13, green) and subcellular markers (red) on day 3. Scale bar: 25 µm

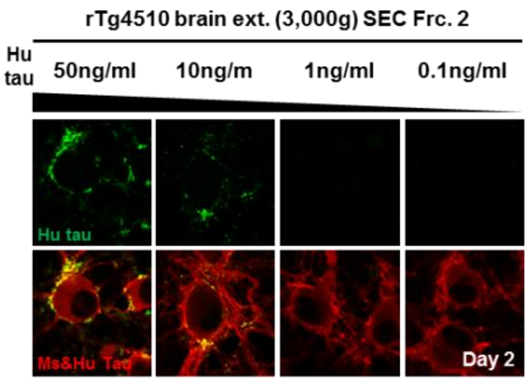

Supplementary Figure 4 Concentration dependency of tau uptake *in vitro*. Primary neurons were incubated with rTg4510 brain extracts (12 months old, PBS-3,000g, 0.1 – 50 ng/ml human tau) and immunostained with human tau specific antibody (Tau13, green) and total (human and mouse) tau antibody (red). Scale bar: 25 µm

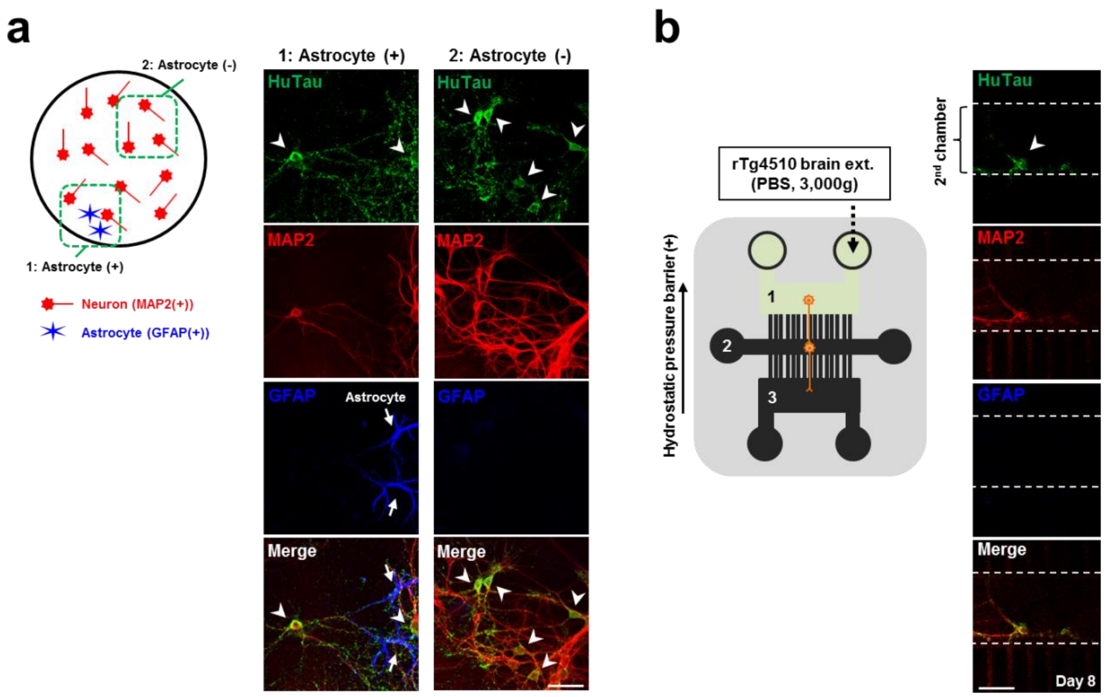

Supplementary Figure 5 Neuronal tau uptake and propagation in the absence of astrocytes. (a, b) Mouse primary neurons were incubated with rTg4510 (12 months old) brain extracts (PBS-3,000g, 500 ng/ml human tau) in a normal culture dish (a) or 3-chamber microfluidic device (b), and immunostained with human tau specific antibody (Tau13, green), MAP2 antibody (red, neuronal marker), and GFAP antibody (blue, astrocyte marker). (a) Neuronal tau uptake in the absence of astrocyte. GFAP positive astrocytes, although infrequent, were found in the primary neuron culture dish (a, 1: Astrocyte (+), arrow). Neuronal tau uptake (arrow head) was detected in both the presence (a, 1: Astrocyte (+)) and absence (a, 2: Astrocyte (-)) of astrocytes. (b) Neuron-to-neuron transfer of tau in the absence of astrocytes. There was no detectable astrocyte contamination in the 2nd chamber of the microfluidic device. rTg4510 brain extract was added to the 1st chamber and human tau positive neuron was detected in the 2nd chamber (day 8) in the absence of astrocyte. Scale bar: 50 µm.

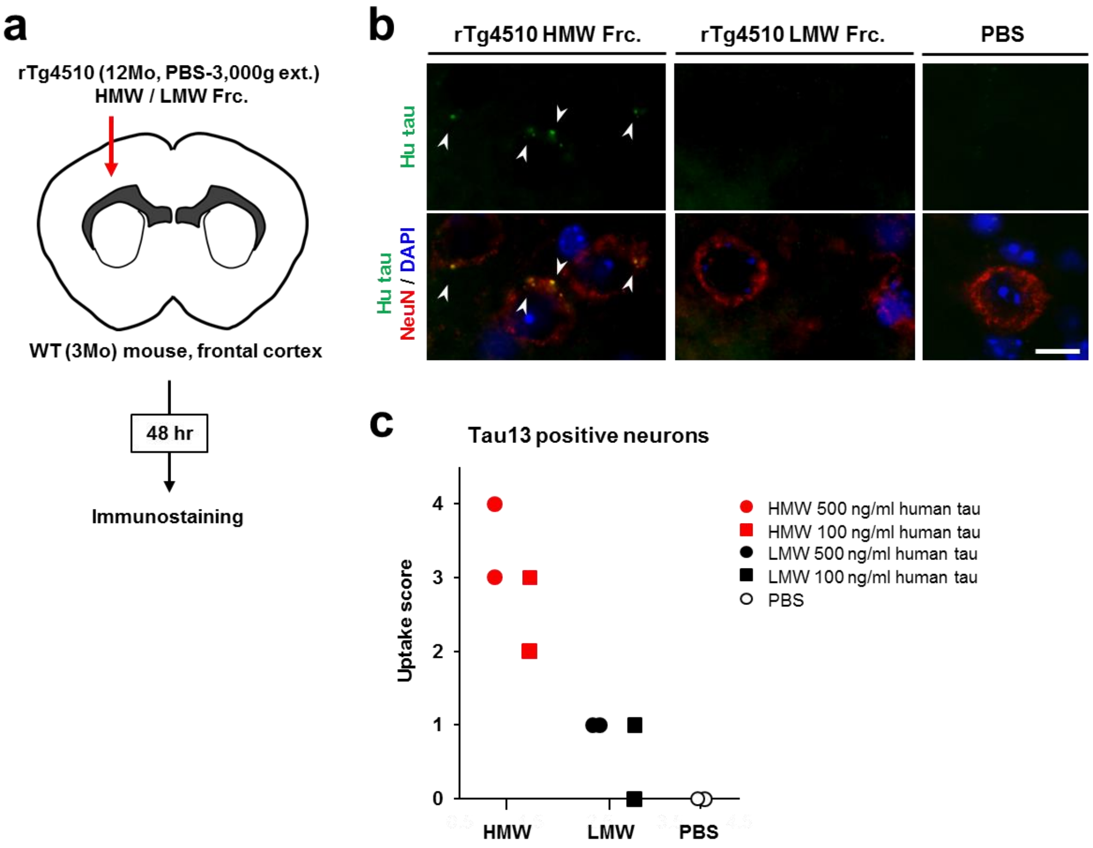

Supplementary Figure 6 HMW tau uptake into neurons *in vivo*. (a) HMW (Frc.2-3) / LMW (Frc.13-14) SEC fractions from rTg4510 brain extract (12 months old, PBS-3,000g, 100 or 500 ng/ml human tau) or PBS were injected into the left frontal cortex of 3-month-old WT mice. (b) 48 hours after injection, brains were collected and immunostained with human tau specific antibody (Tau13, green), anti-NeuN antibody (red, neuronal marker), and DAPI (blue). Human tau positive neurons were detected from mice injected with HMW fraction (arrow heads). (c) Semi-quantitative analysis of human tau positive neurons. Scale bar: 10 μm.

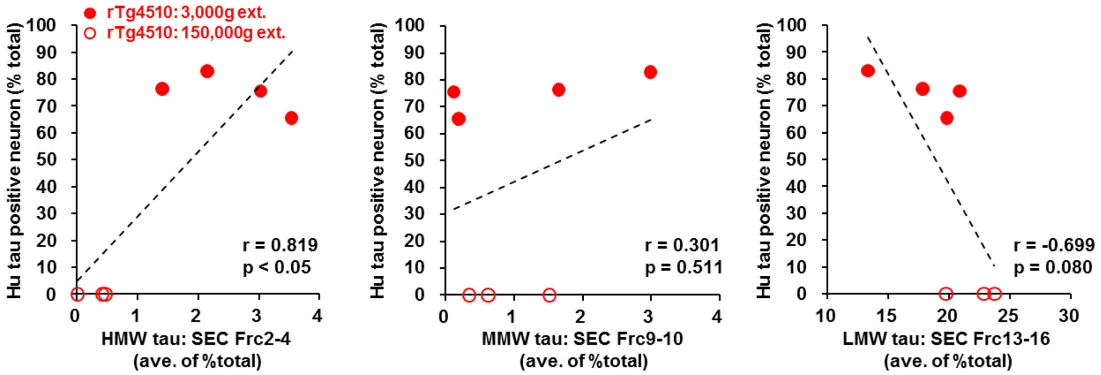

Supplementary Figure 7 Correlations of tau uptake by primary neurons with human tau levels in each SEC-separated fraction. The degree of neuronal tau uptake correlated with HMW tau levels, but not with MMW or LMW tau levels. HMW, high molecular weight; MMW, middle molecular weight; LMW, low molecular weight. 11–13 months old rTg4510 mice were used (n = 7). Pearson correlation analysis.

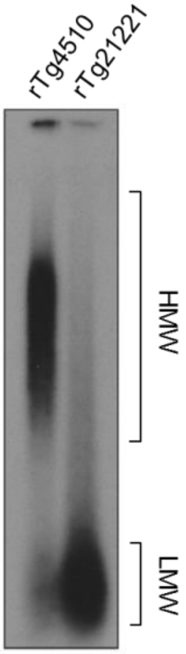

Supplementary Figure 8 SDD-AGE analysis of PBS-soluble brain extracts. (a) SDD-AGE of brain extracts (PBS-3,000g) from rTg4510 and rTg21221 mice (12 months old) demonstrates lack of HMW tau species in rTg21221 mice, although the rTg4510 brain has both HMW and LMW tau. Rabbit polyclonal anti-total tau antibody (#ab64193, Abcam) was used as primary antibody. HMW, high molecular weight; LMW, low molecular weight. SDD-AGE, Semi-denaturing detergent agarose gel electrophoresis.

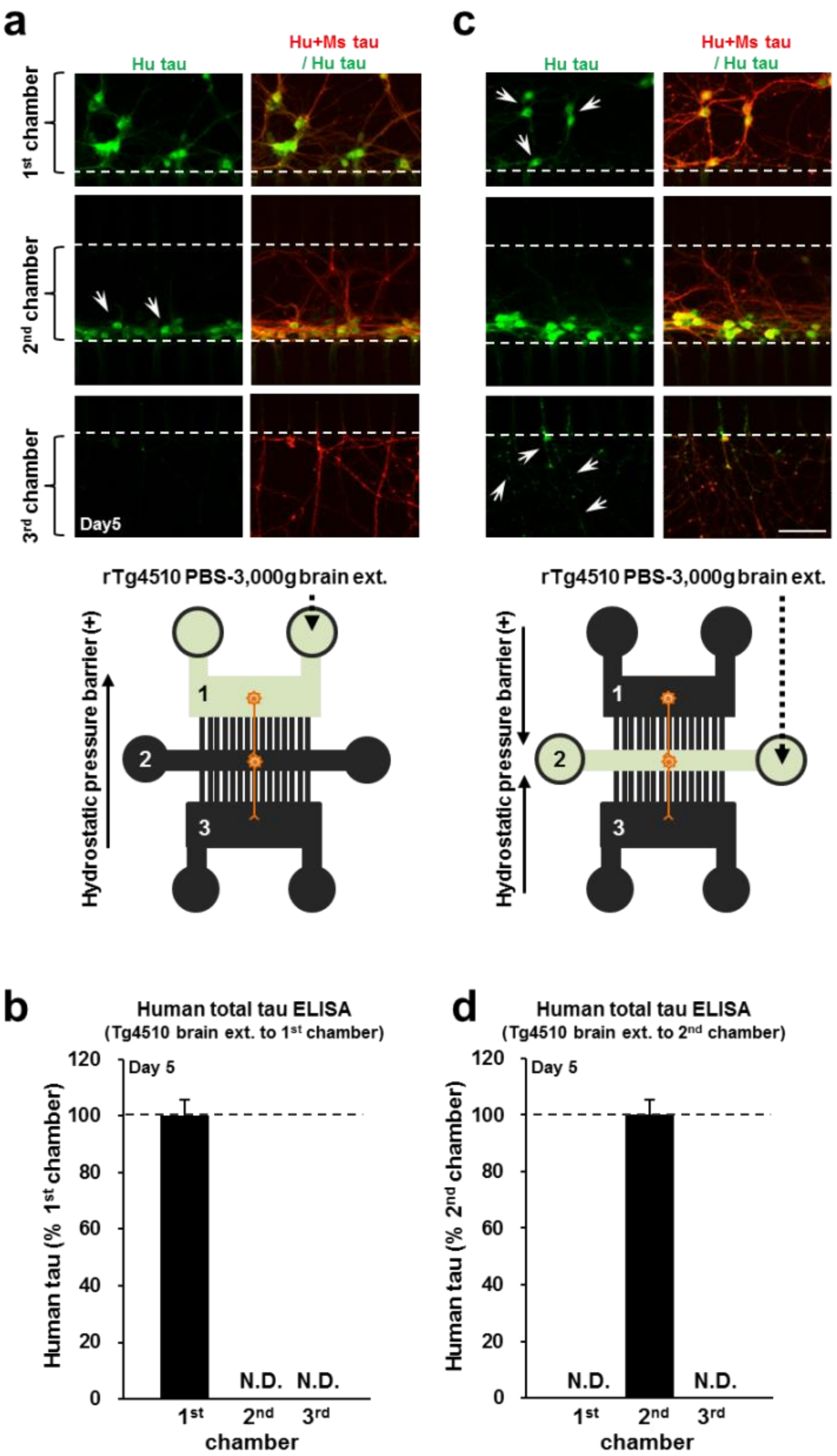

a
Hu tau
Hu+Ms tau / Hu tau
1st chamber
2nd chamber
3rd chamber
Day5
rTg4510 PBS-3,000g brain ext.
Hydrostatic pressure barrier (+)
c
Hu tau
Hu+Ms tau / Hu tau
rTg4510 PBS-3,000g brain ext.
Hydrostatic pressure barrier (+)
b
Human total tau ELISA
(Tg4510 brain ext. to 1st chamber)
Day 5
Human tau (% 1st chamber)
N.D.
N.D.
1st 2nd 3rd
chamber
d
Human total tau ELISA
(Tg4510 brain ext. to 2nd chamber)
Day 5
Human tau (% 2nd chamber)
N.D.
N.D.
1st 2nd 3rd
chamber

Supplementary Figure 9 Anterograde and retrograde tau propagation in a three-chambered microfluidic device. (a, b) Anterograde tau propagation in a 3-chamber microfluidic device. rTg4510 brain extract (12 months old, PBS-3,000g, 500 ng/ml human tau) was added to the 1st chamber. (a) Human tau-positive neurons were detected in the 2nd chamber on day 5 (arrow). (b) Quantification of human tau levels in the culture media collected from the 1st, 2nd, and 3rd chambers on day 5 (human total tau ELISA). There was no detectable level of human tau in the 2nd or 3rd chamber. (*n* = 3) (c, d) Retrograde tau propagation in a 3-chamber microfluidic device. rTg4510 brain extract (12 months old, PBS-3,000g, 500 ng/ml human tau) was added to the 2nd chamber. (c) Human tau-positive neurons and axons were detected in the 1st and 3rd chambers on day 5 (arrow). (d) Quantification of human tau levels in the culture media collected from the 1st, 2nd, and 3rd chambers on day 5 (human total tau ELISA). There was no detectable level of human tau in the 1st or 3rd chamber. (*n* = 3) Diffusion of brain extract between chambers was blocked by a hydrostatic pressure barrier. Scale bar: 50 µm.

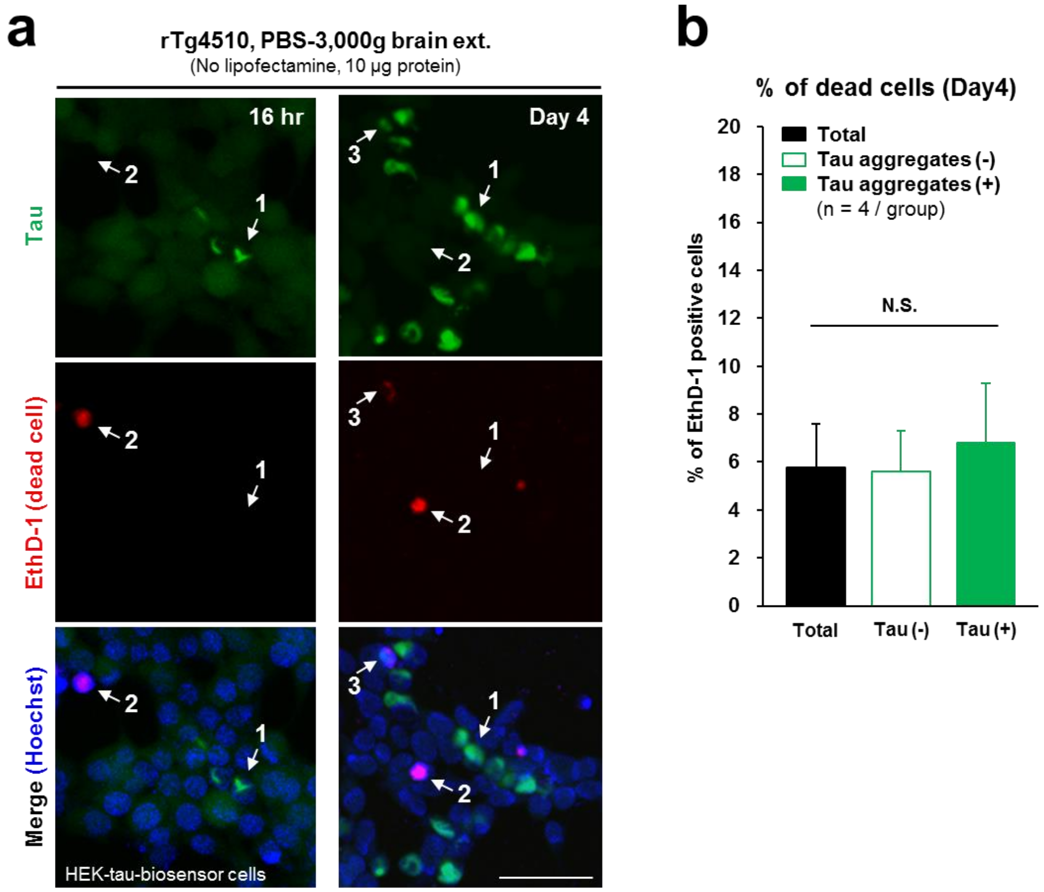

Supplementary Figure 10 Tau uptake and intracellular aggregation do not cause acute cell death. (a, b) Cell viability assay with ethidium homodimer-1 (EthD-1) staining. rTg4510 brain extract (12 months old, PBS-3,000g, 10 µg protein) was transduced into HEK-tau-biosensor cells in the absence of lipofectamine. Cells were stained with EthD-1 (4 µM), which stains the dead cells due to their compromised cell membranes and leaves the healthy cells unstained, and Hoechst 33342 (1 µg/ml) at the time points of 16 hours and day 4. (a) Confocal images at 16 hours (left) and on day 4 (right) shows: tau sensor cells with intracellular tau aggregates, but negative for EthD-1 staining (1), EthD-1 positive dead cells without tau aggregates (2), and EthD-1 positive dead cells with tau aggregates (3). (b) Percentage of EthD-1 positive dead cells in total, tau aggregates negative (Tau (-)), and tau aggregates positive (Tau (+)) cells (day 4). Total number of cells was obtained by Hoechst staining. There was no difference in the percentage of dead cells among groups (n = 4 / group, one-way ANOVA, $F(2, 9) = 0.099$; $P = 0.906$). Scale bar: 50 µm.

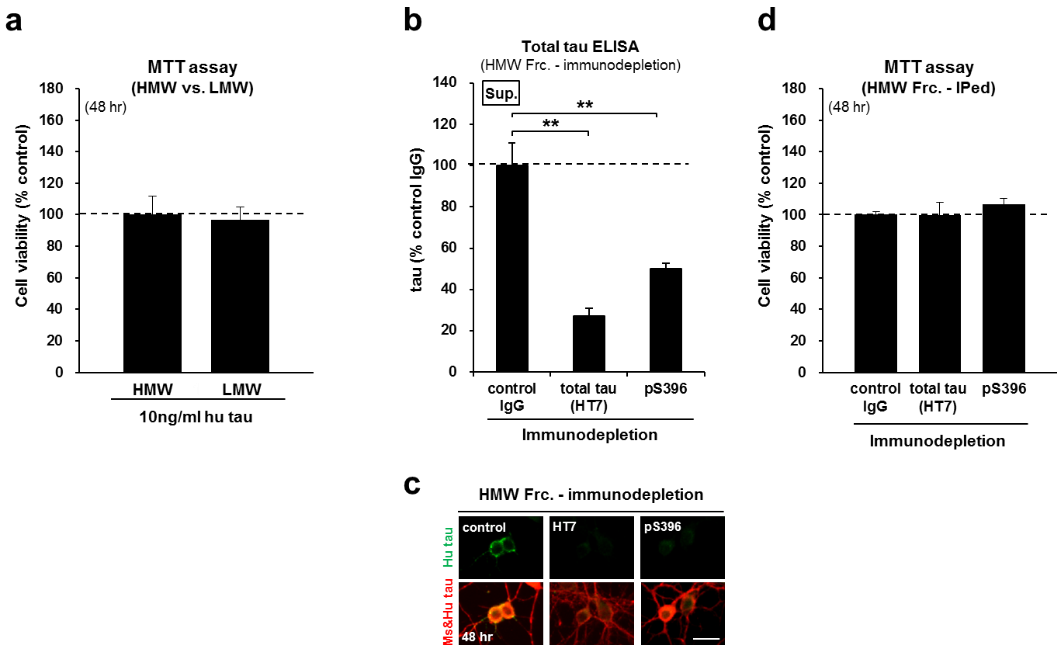

Supplementary Figure 11 Effect of HMW tau on neuronal viability (MTT assay). (a) Mouse primary neurons were incubated with HMW (Frc.2) / LMW (Frc.14) SEC fractions from rTg4510 brain extracts (12 months old, PBS-3,000g, 10 ng/ml human tau). Neuronal viability was measured by MTT assay at 48 hours. There was no difference in MTT-reducing activity between groups (n = 4 / group, P = 0.829, Student's t-test). (b-d) Effect of immunodepletion of HMW tau on neuronal tau uptake and viability. (b) HMW SEC fraction from rTg4510 brain extracts (12 months old, PBS-3,000g, 10 ng/ml human tau) were immunodepleted with total (HT7) or phospho (pS396) tau antibodies (n = 3 / group, $^{**}P < 0.01$, one-way ANOVA and a subsequent Tukey-Kramer test). (c) Mouse primary neurons were incubated with immunodepleted HMW fractions for 48 hours and immunostained with human tau specific antibody (green) and total (human and mouse) tau antibody (red). Immunodepletion with total (HT7) and phospho (pS396) tau antibodies reduced neuronal tau uptake. Scale bar: 20 µm. (d) Immunodepletion with total (HT7) and phospho (pS396) tau antibodies did not alter neuronal MTT-reducing activity at 48 hours (n = 3 / group, P = 0.575, one-way ANOVA).

Full length version of western blots from Figure 2c.

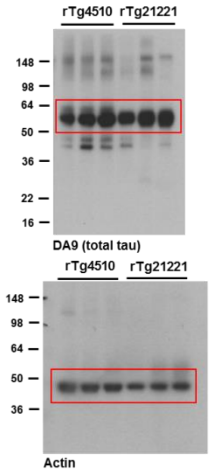

Full length version of western blots from Figure 2d.

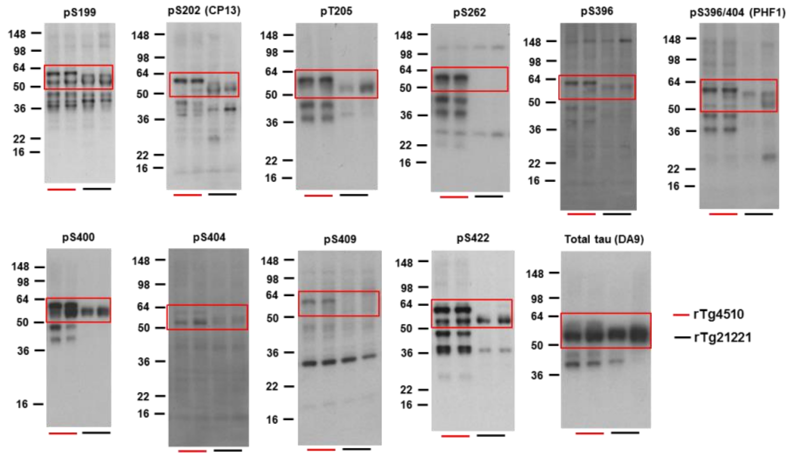

Full length version of western blots from Figure 6i.

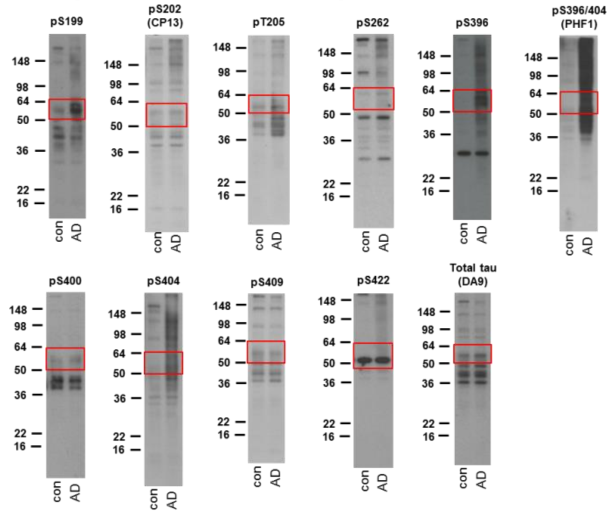

Full length version of western blots from Figure 7d.

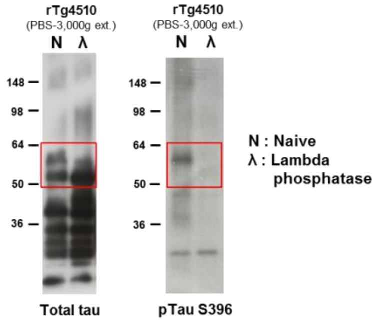

Full length version of western blot from Figure S2c.

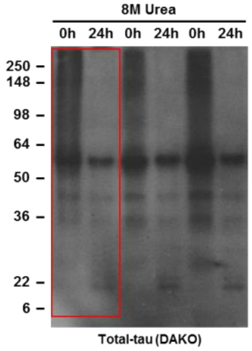

Supplementary Figure 12 Full length versions of western blots.

Characteristics of the subjects with AD and controls used in the study

Case (sample #)	Age at death (years)	Sex	Postmortem-interval (hours)	Diagnosis	Braak stage, CERAD score
AD (#1762)	71	Female	16	AD	VI, C
AD (#1745)	85	Male	24	AD	VI, C
AD (#1683)	83	Male	10	AD	VI, C
AD (#1497)	82	Female	8	AD	VI, C
Control (#1703)	73	Female	20	Control	-
Control (#1669)	86	Male	10	Control	-
Control (#1506)	86	Male	10	Control	-

Supplementary Table 1 Characteristics of the subjects with AD and controls used in the study.

Cases were matched for age (80.3 ± 3.15 (AD) vs. 81.7 ± 4.33 (control) years, $P = 0.796$, $t(5) = -0.273$) and postmortem interval (14.5 ± 3.59 (AD) vs. 13.3 ± 3.33 (control) hours, $P = 0.828$, $t(5) = 0.229$). Mean ± S.E.M., Student’s t-test.
